# Efficacy and acceptability of different probiotic products plus laxatives for pediatric functional constipation: a network meta-analysis of randomized controlled trials

**DOI:** 10.1007/s00431-024-05568-6

**Published:** 2024-05-29

**Authors:** Wei-Chieh Yang, Bing-Syuan Zeng, Chih-Sung Liang, Chih-Wei Hsu, Kuan-Pin Su, Yi-Cheng Wu, Yu-Kang Tu, Pao-Yen Lin, Brendon Stubbs, Tien-Yu Chen, Yen-Wen Chen, Yow-Ling Shiue, Bing-Yan Zeng, Mein-Woei Suen, Chao-Ming Hung, Ming-Kung Wu, Ping-Tao Tseng

**Affiliations:** 1 Department of Pediatrics, Ping An Medical Clinic, Tainan, Taiwan; 2 Department of Internal Medicine, E-Da Cancer Hospital, I-Shou University, Kaohsiung, Taiwan; 3 Department of Psychiatry, Beitou Branch, Tri-Service General Hospital; School of Medicine, National Defense Medical Center, Taipei, Taiwan; 4 Department of Psychiatry, National Defense Medical Center, Taipei, Taiwan; 5 Department of Psychiatry, Kaohsiung Chang Gung Memorial Hospital and Chang Gung University College of Medicine, Kaohsiung, Taiwan; 6 Mind-Body Interface Research Center (MBI-Lab), China Medical University Hospital, Taichung, Taiwan; 7 College of Medicine, China Medical University, Taichung, Taiwan; 8 An-Nan Hospital, China Medical University, Tainan, Taiwan; 9 Department of Sports Medicine, Landseed International Hospital, Taoyuan, Taiwan; 10 Institute of Health Data Analytics & Statistics, College of Public Health, National Taiwan University, Taipei, Taiwan; 11 Department of Dentistry, National Taiwan University Hospital, Taipei, Taiwan; 12 Institute for Translational Research in Biomedical Sciences, Kaohsiung Chang Gung Memorial Hospital, Kaohsiung, Taiwan; 13 Department of Psychological Medicine, Institute of Psychiatry, Psychology and Neuroscience, King’s College London, London, UK; 14 Physiotherapy Department, South London and Maudsley NHS Foundation Trust, London, UK; 15 Positive Ageing Research Institute (PARI), Faculty of Health, Social Care Medicine and Education, Anglia Ruskin University, Chelmsford, UK; 16 Department of Psychiatry, Tri-Service General Hospital, School of Medicine, National Defense Medical Center, Taipei, Taiwan; 17 Institute of Brain Science, National Yang Ming Chiao Tung University, Taipei, 112 Taiwan; 18 Department of Psychology, College of Medical and Health Science, Asia University, 500, Lioufeng Rd., Wufeng Taichung, 41354, Taiwan; 19 Gender Equality Education and Research Center, Asia University, Taichung, Taiwan; 20 Department of Medical Research, Asia University Hospital, Asia University, Taichung, Taiwan; 21 Department of Medical Research, China Medical University Hospital, China Medical University, Taichung, Taiwan; 22 Prospect Clinic for Otorhinolaryngology & Neurology, No. 252, Nanzixin Road, Nanzi District, Kaohsiung City, 81166, Taiwan; 23 Institute of Biomedical Sciences, National Sun Yat-sen University, Kaohsiung, Taiwan; 24 Institute of Precision Medicine, National Sun Yat-sen University, Kaohsiung, Taiwan; 25 Department of Internal Medicine, E-Da Dachang Hospital, I-Shou University, Kaohsiung, Taiwan; 26 Division of General Surgery, Department of Surgery, E-Da Cancer Hospital, I-Shou University, Kaohsiung, Taiwan; 27 School of Medicine, College of Medicine, I-Shou University, No. 1, Sec. 1, Xuecheng Rd., Dashu Dist., Kaohsiung City, 840301, Taiwan

**Keywords:** Probiotics, Constipation, Network meta-analysis, Pediatrics, Family medicine

## Abstract

The prevalence of pediatric constipation ranges from 0.7 to 29.6% across different countries. Functional constipation accounts for 95% of pediatric constipation, and the efficacy of pharmacotherapy is limited, with a success rate of 60%. Several randomized controlled trials (RCTs) have shown the benefits of probiotic supplements in treating this condition. However, the reported strains of probiotics varied among the RCTs. We aimed to compare the efficacy and acceptability of different probiotic supplements for pediatric functional constipation. The current frequentist model–based network meta-analysis (NMA) included RCTs of probiotic supplements for functional constipation in children. The primary outcome was changes in bowel movement or stool frequency; acceptability outcome was all-cause discontinuation. Nine RCTs were included (*N* = 710; mean age = 5.5 years; 49.4% girls). Most probiotic products, used either alone or combined with laxatives, were associated with significantly better improvement in bowel movement or stool frequency than placebo/control. Protexin plus laxatives (standardized mean difference (SMD) = 1.87, 95% confidence interval (95% CI) = 0.85 to 2.90) were associated with the greatest improvement in bowel movement or stool frequency among all the investigated probiotic products. For the single probiotic interventions, only *Lactobacillus casei rhamnosus* Lcr35 was associated with significant efficacy compared to placebo/control treatments (SMD = 1.37, 95% CI: 0.32 to 2.43). All the investigated probiotic products had fecal incontinence and patient drop-out rates similar to those of placebo/control treatments.

*Conclusion*: The results of our NMA support the application of an advanced combination of probiotics and laxatives for pediatric functional constipation if there is no concurrent contraindication.

*Registration*: PROSPERO (CRD42022298724).

**Table:** 

**What is Known:** *• Despite of the high prevalence of pediatric constipation, which ranges from 0.7% to 29.6%, the efficacy of pharmacotherapy is limited, with a success rate of 60%. Several randomized controlled trials (RCTs) have shown the benefits of probiotic supplements in treating this condition. However, the reported strains of probiotics varied among the RCTs. The widely heterogeneous strains of probiotics let the traditional meta-analysis, which pooled all different strains into one group, be nonsense and insignificant.*	
**What is New:** *• By conducting a comprehensive network meta-analysis, we aimed to compare the efficacy and acceptability of different strains of probiotic supplements for pediatric functional constipation. Network meta-analysis of nine randomized controlled trials revealed that the most probiotic products, used either alone or combined with laxatives, were associated with significantly better improvement in bowel movement or stool frequency than placebo/control. Protexin plus laxatives was associated with the greatest improvement in bowel movement or stool frequency among all the investigated probiotic products. For the single probiotic interventions, only Lactobacillus casei rhamnosus Lcr35 was associated with significant efficacy compared to placebo/control treatments. All the investigated probiotic products had fecal incontinence and patient drop-out rates similar to those of placebo/control treatments.*

**Supplementary Information:**

The online version contains supplementary material available at 10.1007/s00431-024-05568-6.

## Introduction

Pediatric constipation is a frequently ignored but common health problem worldwide; it is one of the reasons for frequent emergency room visits and results in high medical costs [1]. The prevalence of pediatric constipation varies in different countries and ranges from 0.7 to 29.6% (median 12.0%) [2]. Functional constipation, which accounts for 95% of chronic constipation in children, refers to constipation without definite organic lesions [3]. The potentially underlying pathophysiology of functional constipation included including stool withholding behavior, anorectal dysfunctions, diet, physical activity, alteration of microbiome, gastrointestinal motility disorder, genetic predisposition, and psychological issues [2, 4]. Among them, the stool withholding behavior is one of the main mechanisms in pediatric functional constipation (around 37–91%) [4]. The untreated functional constipation would lead to fecal impaction, retentive fecal incontinence, loss of appetite, and tendency to urinary infections. Although the pediatric functional constipation is a multifactorial disease, and the pathophysiology remains unclear, one of the main hypothetical pathophysiologies of functional constipation is dysbiosis in the gut microbiota [5]. Children with constipation were found to have fewer interstitial cells of Cajal, which are the major cells determining gut motility [6]. There is also a hypothesis of reduced short-chain fatty acids, which are metabolites of intestinocolonal flora [7].

Laxatives, including osmotic laxatives (for example, lactulose and magnesium hydroxide), fecal softeners (for example, mineral oil), stimulant laxatives (for example, bisacodyl, senna, and sodium picosulfate), and rectal laxatives/enemas (for example, sodium docusate and sodium phosphate) [3], are the regimens of choice for pediatric functional constipation; however, 40% of children do not respond adequately to this traditional medical treatment [8]. Importantly, 45.8 to 63.8% of parents have concerns about laxative dependence after long-term use of laxatives in their children [9]. Another concern is the potential association between long-term laxative use and the risk of electrolyte imbalance or dehydration [10]. Therefore, determining alternatives to manage functional constipation has become an important issue in clinical pediatric practice.

Probiotics have become one potential choice to manage functional constipation in children because they could alter the dysbiosis in the gut microbiota in children with functional constipation, which is one of the main hypothesized pathophysiologies of functional constipation. Nevertheless, the previous clinical trials of probiotics provided controversial results. The efficacy and clinical indication of different probiotics varied widely across different strains of probiotics [11]. Therefore, in previous pairwise meta-analyses [12, 13], after pooling different strains of probiotics into one group, the authors concluded that probiotics were ineffective in the management of pediatric constipation. However, because the efficacy and clinical indication of different probiotics varied widely across different probiotic strains, the unsatisfactory result of one strain did not indicate the efficacy of another strain of probiotics [11].

A well-designed network meta-analysis (NMA) has the merit to multiply compared to the efficacy between individual strains of probiotics for the management of functional constipation in children. Evidence from NMA can thus inform further research and provide evidence to support a new rationale for future large-scale trials [14]. The current study had the primary aim of comparing different probiotic supplementation treatments with respect to their effectiveness and their acceptability in children (i.e., age less than 18 years old) with functional constipation.

## Methods

### General guidelines applied in the current study

Following PRISMA2020 guidelines (eTable 1) [15] and AMSTAR2 (A MeaSurement Tool to Assess systematic Reviews) [16], we conducted this frequentist-based NMA. The Institutional Review Board of the Tri-Service General Hospital, National Defense Medical Center, had approved this NMA (TSGHIRB No. B-109-29), which had also been registered on PROSPERO (CRD42022298724).

### Search strategy and selection criteria

The detailed search strategy was listed in eTable 2. To include as many articles as possible, we manually reached for potentially eligible studies cited in review articles and pairwise meta-analyses. Furthermore, we did not set any filter in the electronic databases regarding the language restriction.

### Selection criteria

We applied PICO design as (1) patients: children with functional constipation without specific organic, neurologic, or psychiatric origin; (2) intervention: probiotic supplement; (3) comparator: waiting-list, placebo-control, or active-control; and (4) outcome: changes in bowel movement or stool frequency.

Therefore, the inclusion criteria were as follows: (1) clinical RCTs with either waiting lists, placebo controls, or active controls and (2) recruitment of children with functional constipation (i.e., children were defined as participants less than 18 years old); (3) children with constipation due to other definite origin (i.e., specific organic, neurologic, or psychiatric origin) were excluded; and (4) the efficacy of probiotics (or products containing probiotics) on bowel movement or stool frequency was investigated.

Studies were excluded if they (1) are non-RCTs, (2) not regarding outcomes of interest, or (3) were not specific to children with functional constipation diagnosis (i.e., RCTs with diarrhea, irritable bowel syndrome, organic constipation, or comorbidities with neuropsychiatric disease were excluded). In situations in which the same set of sample sources had been used by multiple studies, we would select the most informative study.

### Data extraction

Two authors independently screened the studies and extracted the data of interest from the articles. If encountering inconsistent opinions, the corresponding author adjudicated the disagreement. If the manuscripts lacked relevant data, we contacted the corresponding authors or coauthors to obtain the originally used data.

### Outcomes

Because there had been a report demonstrating a high placebo effect on the “subjective” outcome (i.e., patients gave a self-report of satisfactory remission using a single question, for example, “How do you feel about your constipation symptoms now?”) in the RCTs of therapy for functional constipation [17], we did not choose those subjective outcome to be our outcome. In addition, we did not choose “response/successful rate” to be our outcome because the definition of “response/successful” varied widely across the RCTs. Rather, we chose an objective outcome (i.e., bowel movement or stool frequency) as our primary outcome. The definition of bowel movement or stool frequency was the number of bowel movements/stool passages per fixed time period (which varied across the included RCTs). The safety profile was set to be the rate of fecal incontinence. Acceptability would be counted as the drop-out rate, which would be considered as a participant leaving the study before the end of the study for any reason.

### Cochrane risk of bias tool

Two authors independently evaluated the risk of bias for each domain per the Cochrane risk of bias tool [18].

### Statistical analysis

We performed NMA on STATA version 16.0 (StataCorp LLC, College Station, TX, USA). We estimated the summary standardized mean difference (SMD) with their corresponding 95% confidence intervals (95%CIs) for the estimated effect size of continuous outcomes. We estimated the summary odds ratio (OR) with their corresponding 95%CIs for categorical outcomes. For statistics necessity, we followed the 0.5 zero-cell correction method in our meta-analytic procedure for the categorical data. However, in situation of zero event in both arm in one study, we would not use such correction because of the potential bias to contribute statistical bias [19, 20]. We used the frequentist model of NMA to compare the effect sizes (ES) between studies with the same intervention. Heterogeneity among the included studies would be analyzed by the tau value.

The direct- and indirect-evidence in this NMA would be calculated and analyzed under the generalized linear mixed model [21]. The package program in the STATA for the current NMA was the *mvmeta* command [22]. Furthermore, the restricted maximum likelihood method would be applied to investigate the between-study variance [23]. To hypothesize the specific efficacy of the probiotics, we compared the efficacy between the “treatment arm with only probiotics” and “placebo or laxatives” in the subgroup analysis. Furthermore, we applied the surface under the cumulative ranking curve (SUCRA) to rank the relatively superiority between the experimental arms on the indicated outcomes [24]. Finally, the inconsistencies among the current NMA would be investigated with the loop-specific approach, node-splitting method, and design-by-treatment model [25]. We applied the GRADE ratings to examine the quality of evidence [26, 27].

## Results

**Fig. 1:**
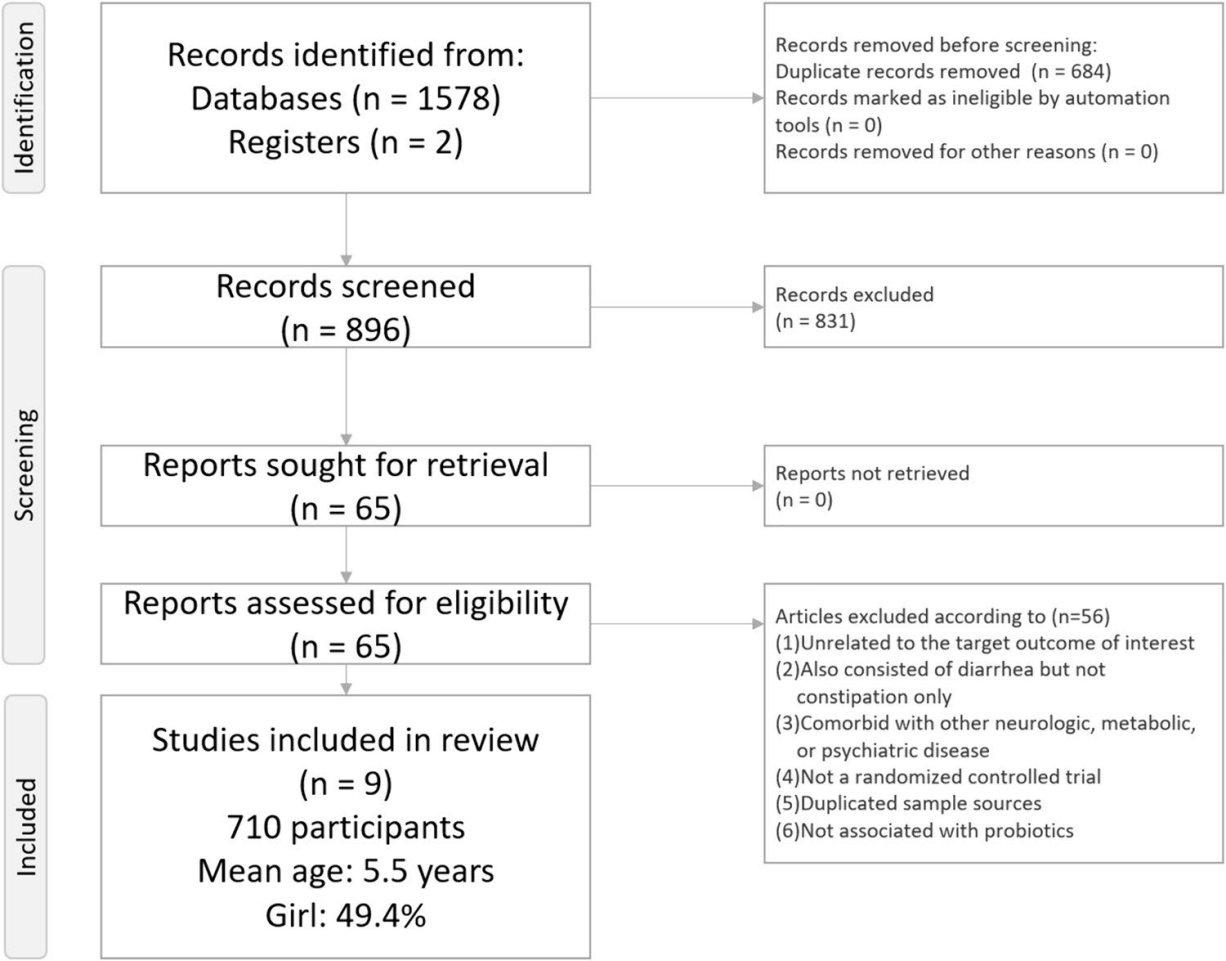
Flowchart illustrating the procedure of the present network meta-analysis

**Fig. 2:**
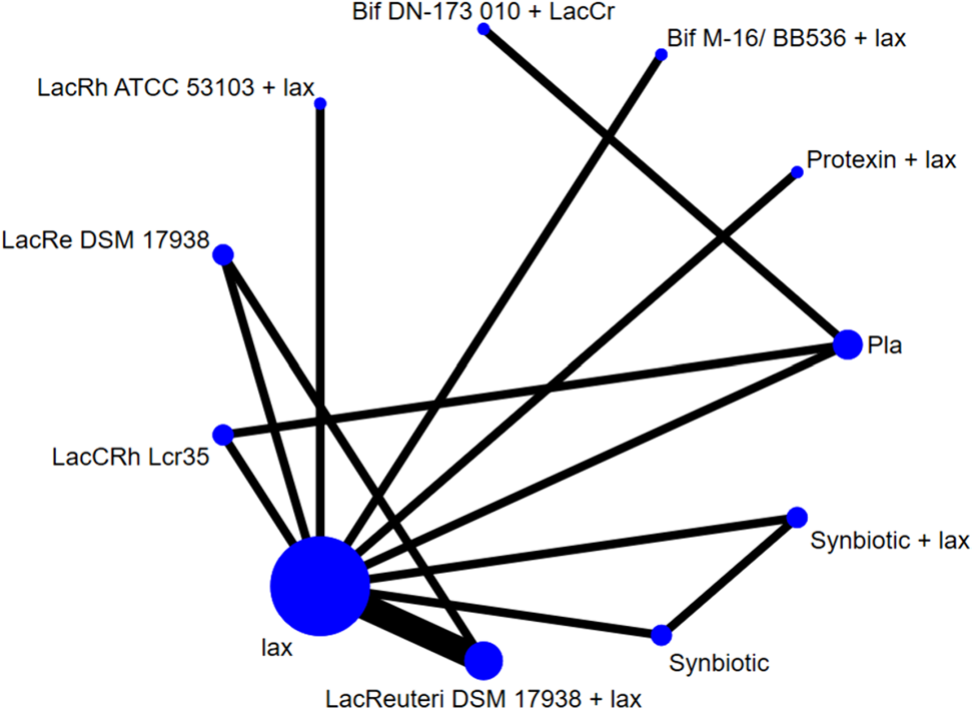
Overall network structure of the current network meta-analysis for the primary outcome of changes in bowel movement or stool frequency. The lines between nodes represent direct comparisons in various trials, and the size of each circle is proportional to the number of participants receiving each specific treatment. The thickness of the lines is proportional to the number of trials connected to the network

After the initial screening procedure, 65 articles were selected into the full-text screen stage (Fig. 1). However, 56 articles were excluded due to ineligibility (Fig. 1; eTable 3). Among them, one RCT [28] was excluded because the placebo effect in that RCT was unusually higher than the average in other RCTs of functional constipation (70% vs. 18.31% to 20.35%) [17]. Finally, we included nine articles in the NMA (eTable 4) [11, 29–36]. The overall network structure of the treatment arms is illustrated in Fig. 2.

### Detailed information of the included studies

The nine RCTs had 710 participants in total. The mean age was 5.5 years (range 2.9 to 7.2 years), and 49.4% of participants were girls (range 44.2 to 69.7%). The mean treatment duration was 6.1 weeks (range 3 to 12 weeks). The average total study duration (i.e., treatment plus follow-up duration) was 8.5 weeks (range 4 to 24 weeks).

### Primary outcome: changes in bowel movement or stool frequency

**Fig. 3:**
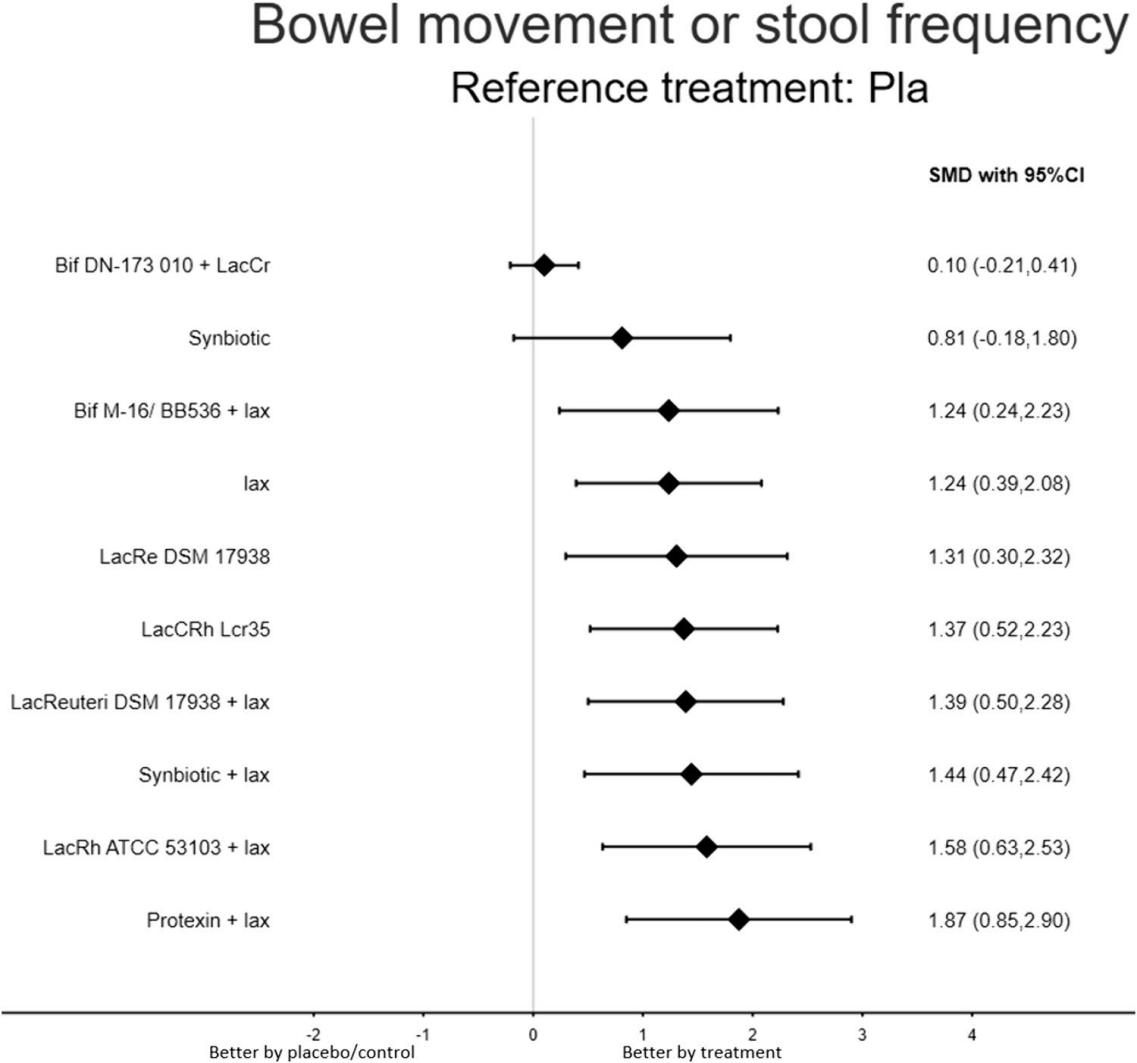
When the effect size was > 0 (presented as the standardized mean difference), the specified treatment yielded a better improvement in bowel movement or stool frequency than its corresponding sham/control treatment. Abbreviations: 95% CI, 95% confidence interval; Bif DN-173 010 + LacCr, probiotic (Bifidobacteria lactis DN-173 010 + Lactococcus cremoris); Bif M-16/ BB536 + lax, probiotic mixture (Bifidobacteria breve + longum) + laxative; ES, effect size; LacCRh Lcr35, Lactobacillus casei rhamnosus Lcr35; LacRe DSM 17938, Lactobacillus reuteri DSM 17938; LacReuteri DSM 17938 + lax, Lactobacillus reuteri DSM 17938 + laxative; LacRh ATCC 53103 + lax, Lactobacillus rhamnosus GG (ATCC 53103) + laxative; lax, laxative; NMA, network meta-analysis; OR, odds ratio; Pla, placebo/control; Protexin + lax, Protexin (Lactobacillus casei PXN 37, Lactobacillus rhamnosus PXN 54, Streptococcus thermophiles PXN 66, Bifidobacterium breve PXN 25, Lactobacillus acidophilus PXN 35, Bifidobacterium infantis (child specific) PXN 27, and Lactobacillus bulgaricus PXN 39) + laxative; RCT, randomized controlled trial; SMD, standardized mean difference; SUCRA, surface under the cumulative ranking curve; synbiotic + lax: synbiotic (L. casei, L. rhamnosus, S. thermophilus, B. breve, L. acidophilus, B. infantis, fructooligosaccharide) + laxative

The results revealed that several probiotics products were associated with significantly better improvement in bowel movement or stool frequency than the placebo/control treatment, including Protexin plus laxatives, Lactobacillus rhamnosus GG ATCC 53103 plus laxative, Synbiotic plus lax, Lactobacillus reuteri DSM 17938 plus laxative, Lactobacillus casei rhamnosus Lcr35, Lactobacillus reuteri DSM 17938, and probiotic mixture (Bifidobacteria breve + longum) plus laxative. Among the investigated probiotics, only two species did not achieve statistical significance (Table 1; Figs. 2 and 3). Furthermore, the Protexin + lax gave the most improvement based on the SUCRA statistics (eTable 5A).

**Table 1:** League table of the improvement in bowel movement or stool frequency

Protexin + lax							*0.64 (0.06,1.22)			
0.29 (−0.43, 1.02)	LacRh ATCC 53103 + lax						0.35 (−0.09, 0.78)			
0.43 (−0.33, 1.19)	0.14 (−0.51, 0.79)	Synbiotic + lax					0.21 (−0.28, 0.69)	*0.63 (0.15, 1.12)		
0.48 (−0.16, 1.13)	0.19 (−0.32, 0.70)	0.05 (−0.51, 0.61)	LacReuteri DSM 17938 + lax		0.03 (−0.60, 0.65)		0.15 (−0.12, 0.43)			
0.50 (−0.37, 1.38)	0.21 (−0.58, 0.99)	0.07 (−0.75, 0.88)	0.02 (−0.69, 0.73)	LacCRh Lcr35			0.14 (−0.52, 0.79)			*1.37 (0.52, 2.23)
0.57 (−0.23, 1.37)	0.28 (−0.43, 0.98)	0.14 (−0.60, 0.87)	0.08 (−0.47, 0.64)	0.07 (−0.79, 0.92)	LacRe DSM 17938		0.02 (−0.60, 0.63)			
0.64 (−0.15, 1.42)	0.35 (−0.34, 1.03)	0.21 (−0.51, 0.92)	0.15 (−0.44, 0.75)	0.14 (−0.70, 0.98)	0.07 (−0.69, 0.84)	Bif M-16/ BB536 + lax	0.00 (−0.53, 0.53)			
*0.64 (0.06, 1.22)	0.35 (−0.09, 0.78)	0.21 (−0.28, 0.69)	0.15 (−0.12, 0.43)	0.14 (−0.52, 0.79)	0.07 (−0.48, 0.62)	−0.00 (−0.53, 0.53)	lax	0.43 (−0.08, 0.94)		*1.24 (0.39, 2.08)
*1.06 (0.29, 1.84)	*0.77 (0.10, 1.44)	*0.63 (0.15, 1.12)	*0.58 (0.00, 1.16)	0.56 (−0.27, 1.39)	0.50 (−0.26, 1.25)	0.43 (−0.31, 1.16)	0.43 (−0.08, 0.94)	Synbiotic		
*1.77 (0.70, 2.84)	*1.48 (0.48, 2.48)	*1.34 (0.32, 2.36)	*1.29 (0.35, 2.23)	*1.27 (0.36, 2.18)	*1.20 (0.15, 2.26)	*1.13 (0.09, 2.18)	*1.13 (0.23, 2.03)	0.71 (−0.33, 1.74)	Bif DN-173 010 + LacCr	0.10 (−0.21, 0.41)
*1.87 (0.85, 2.90)	*1.58 (0.63, 2.53)	*1.44 (0.47, 2.42)	*1.39 (0.50, 2.28)	*1.37 (0.52, 2.23)	*1.31 (0.30, 2.32)	*1.24 (0.24, 2.23)	*1.24 (0.39, 2.08)	0.81 ( 0.18, 1.80)	0.10 (−0.21, 0.41)	Pla

Pairwise (upper-right portion) and network (lower-left portion) meta-analysis results are presented as estimate effect sizes for the outcome of improvement in bowel movement or stool frequency. Interventions are reported in order of mean ranking of bowel movement or stool frequency improvement, and outcomes are expressed as standardized mean difference (SMD) (95% confidence intervals). For the pairwise meta-analyses, SMD of more than 0 indicate that the treatment specified in the row got more improvement than that specified in the column. For the network meta-analysis (NMA), SMD of more than 0 indicate that the treatment specified in the column got more improvement than that specified in the row. Bold results marked with asterisk indicate statistical significance

*95% CI* 95% confidence interval, *Bif DN-173 010* + *LacCr* probiotic (Bifidobacteria lactis DN-173 010 + Lactococcus cremoris), *Bif M-16/ BB536* + *lax* probiotic mixture (Bifidobacteria breve + longum) + laxative, *ES* effect size, *LacCRh Lcr35* Lactobacillus casei rhamnosus Lcr35, *LacRe DSM 17938* Lactobacillus reuteri DSM 17938, *LacReuteri DSM 17938* + *lax* Lactobacillus reuteri DSM 17938 + laxative, *LacRh ATCC 53103* + *lax* Lactobacillus rhamnosus GG (ATCC 53103) + laxative, *lax* laxative; *NMA* network meta-analysis, *OR* odds ratio, *Pla* placebo/control, *Protexin* + *lax* Protexin (Lactobacillus casei PXN 37, Lactobacillus rhamnosus PXN 54, Streptococcus thermophiles PXN 66, Bifidobacterium breve PXN 25, Lactobacillus acidophilus PXN 35, Bifidobacterium infantis (child specific) PXN 27, and Lactobacillus bulgaricus PXN 39) + laxative, *RCT* randomized controlled trial, *SMD* standardized mean difference, *SUCRA* surface under the cumulative ranking curve, *Synbiotic* + *lax* synbiotic (*L. casei*, *L. rhamnosus*, *S. thermophilus*, *B. breve*, *L. acidophilus*, *B. infantis*, fructooligosaccharide) + laxative

### Primary outcome: changes in bowel movement or stool frequency — subgroup of treatment arm with only probiotics vs. placebo or laxatives

To hypothesize the specific efficacy of probiotics, we compared the efficacy between the “treatment arm with only probiotics” and “placebo or active controls (i.e., laxatives).” In this subgroup analysis, and we found that only LacCRh Lcr35 (SMD = 1.37, 95%CIs = 0.32 to 2.43) and laxatives (SMD = 1.24, 95%Cis = 0.09 to 2.38) provided significantly better improvement in bowel movement or stool frequency than placebo/control (eTable 6A; eFigs. 1A and 2A). The LacCRh Lcr35 gave the most improvement based on the SUCRA statistics (eTable 5B).

### Safety profile: rate of fecal incontinence

No significant differences in the rates of fecal incontinence between the investigated interventions had been detected (eTables 5C and 6B; eFigs. 1B and 2B).

### Acceptability calculated with the drop-out rate

No significant differences in drop-out rates between the investigated interventions had been detected (eTables 5D and 6C; eFigs. 1C and 2C).

### Risk of bias, quality of evidence, and publication bias

Overall 76.2% (48/63 items), 20.6% (13/63 items), and 3.2% (2/63 items) of the investigated items revealed low, unclear, and high risks of bias, respectively. The unclear reporting of allocation concealment resulted in the risk of bias (eFig. 3A, B).

The generally symmetric funnel plots of publication bias and Egger’s regression findings suggest no significant publication bias in this study (eFig. 4A–F). There had been no significant inconsistency or heterogeneity detected, either (eTables 7 and 8). The quality of evidence of most comparisons revealed low to medium quality according to GRADE ratings (eTable 9).

## Discussion

The main finding of this NMA is that the most investigated probiotic products in the NMA, either with or without laxatives, provided significantly better improvement in bowel movement or stool frequency than placebo/control treatment. Among them, Protexin + lax provided the greatest improvement in bowel movement or stool frequency among all the investigated probiotic treatments. If we focused on treatment arms with a single probiotic intervention, only LacCRh Lcr35 was associated with significant efficacy. In addition, all the investigated probiotic products had similar rates of fecal incontinence and drop-out rates as placebo/control treatments.

The potentially beneficial effect of the probiotic products in bowel movement/stool frequency noticed in this NMA could be supported by the previous reports. To be specific, several reports have supported the use of probiotic supplementation in children with functional constipation. This rationale of probiotic supplementation was developed based on dysbiosis in the intestinal flora in functionally constipated children compared to healthy persons [5], such as lower *Lactobacillus* spp. [37], *Alistipes* spp., and *Ruminococcus* spp. [38] and increased *Bacteroides* spp., *Parabacteroides* spp., and *Bifidobacterium longum* [38]. Additionally, the reduction of microbial-derived metabolites (i.e., short-chain fatty acids) [7], whose products could promote intestinal motility through the Gpr41 receptor [39], might be another potential etiology of functional constipation. Probiotic supplementation improved short-chain fatty acid production and enhanced colon motility and colonic transit time [34]. In addition to the hypothesis focused on short-chain fatty acids, lowering colonic pH by probiotic supplementation is believed to normalize intestinal function [40]. However, none of these hypotheses could be completely confirmed in the physiopathology of functional constipation because contradictory evidence from breastfeeding infants has been observed [41].

Another important finding was that LacCRh Lcr35 provided significantly better improvement in bowel movement or stool frequency than the placebo/control treatment in the subgroup analysis of the treatment arm with only probiotics vs. placebo or laxatives. This finding could support the aforementioned dysbiosis hypothesis. Specifically, supplementation with Lactobacillus strains in germ-free animals could switch their abnormal migrating motor complexes to nearly normal function [42]. The majority of the efficacy of LacCRh Lcr35 comes from its ability to adhere human intestinal cell lines [43], its ability to maintain colonization after oral consumption, and its antibacterial activity against pathogens [44]. However, although it has been commercially used to manage acute diarrhea for more than 20 years, the optimal dosage of LacCRh Lcr35 to manage pediatric functional constipation remains unclear. Therefore, future large-scale RCTs addressing the optimal dosage of LacCRh Lcr35 are warranted.

The findings of this NMA remarked the recommendations in the previous clinical guidelines of European Society for Paediatric Gastroenterology, Hepatology and Nutrition (ESPGHAN) and North American Society For Pediatric Gastroenterology, Hepatology and Nutrition (NASPGHAN) [3]. To be specific, both the main part of the NMA including all investigated interventions (i.e., probiotics with/without laxatives) and the subgrouping of treatment arms with the single intervention of probiotics (i.e., probiotics without laxatives) revealed that probiotic supplementation was not significantly superior to laxatives only in children with functional constipation. However, our NMA provided an important point that all the investigated treatments of the combination of probiotics and laxatives provided significantly better improvement in bowel movement/stool frequency than the placebo/control treatments. Furthermore, although it did not achieve statistical significance, the combination of probiotics and laxatives was ranked as relatively superior to the use of laxatives only. All the investigated treatments with a combination of probiotics and laxatives had safety and drop-out rates similar to those of placebo/control treatments. These findings echo the findings in a previous RCT [11]. Based on the aforementioned results, we might recommend the application of an advanced combination of probiotics and laxatives for pediatric functional constipation if there is no concurrent contraindication.

## Limitations

Our NMA has some limitations to be addressed. First, although we had tried our best to reduce the heterogeneity of the current NMA by restricting the inclusion criteria to exclude those with severe comorbid neurologic, psychiatric, or metabolic disorders, the results of this NMA might still be confounded by potential heterogeneity between studies with respect to participant characteristics, such as participant age, diagnosis criteria, concomitant medication, and trial duration. In addition, since the laxatives might vary among the included RCTs, the treatment node of “laxatives” might have theoretically potential inconsistency or heterogeneity within it. The treatment node of “laxatives” consisted of different mechanisms, such as lactulose, macrogol, MgO, and liquid paraffin. Some of these laxatives might have potential effects on the gut microbiota composition [45]. To explore this, we had arranged further inconsistency and heterogeneity test (eTable 7) to investigate the potential existence of inconsistency/heterogeneity, which revealed no significance detected. Also, since the types of laxatives varied, we could not arrange dosage comparison regarding the bias by the concomitant laxatives. In addition, although the effect of probiotic products plus laxatives was statistically superior to the use of laxatives only, we could not completely rule out the possibility that the effect of probiotic products plus laxatives may result from only the laxatives. Furthermore, although no statistically significant inconsistency detected, the probiotics arm consists of different probiotic strains, which might contributed to unexpected bias to the results. Second, we recognized the exited small size studies among the recruited RCTs. However, since this field consisted of few RCTs, our comparison between different treatments based on these RCTs would allow us to integrate findings on the effectiveness of different probiotics in children with functional constipation. Third, we could not take the potential confounding factors of diet, lifestyle habits, genetics, and other factors into consideration in the current NMA. These factors had not been fully disclosed in the previous RCTs. Fourth, not all the included RCTs had applied a placebo control, and a placebo effect could therefore have affected their findings [17]. Fifth, although the probiotics plus laxatives provided significantly better improvement in the primary outcome than the placebo/control group, the additive effect of probiotics to the laxatives was relatively small. In addition, although we could derived network comparison through “direct- and indirect-evidence” based on the merits of NMA, there were still few of RCTs providing direct comparisons between some specific experimental arms, including LacCRh Lcr35 vs placebo/controls. Finally, because the average overall study duration among the included RCTs was relatively short (8.5 weeks with a range of 4 to 24 weeks), we could not evaluate the potential risk of relapse after the end of probiotic supplementation. According to a previous report, 15% of children with functional constipation relapse within 3 years of follow-up [46]. Future studies with longer follow-up periods are thus warranted.

## Conclusion

Our NMA showed that the most investigated probiotic products, especially when used in combination with laxatives, provided significantly better improvement in bowel movement/stool frequency than the placebo/control treatments. Only two of the investigated probiotics did not achieve statistical significance. Furthermore, Protexin + lax exerted the most improvement in bowel movement/stool frequency among all the investigated probiotic products. However, if we focused on treatment arms with a single probiotic intervention, only LacCRh Lcr35 was considered to be an intervention with significant efficacy. Finally, all interventions had fecal incontinence and drop-out rates similar to those of placebo/control treatments. The results of our NMA support the rationale of applying an advanced combination of probiotics and laxatives for pediatric functional constipation if there is no concurrent contraindication.

### Supplementary Information

Below is the link to the electronic supplementary material.

Supplementary file1 (PPTX 745 kb)

Supplementary file2 (DOCX 161 kb)

## Data Availability

No datasets were generated or analysed during the current study.

## Declarations

### Disclaimer

The views expressed in this publication are those of the authors and not necessarily those of the acknowledged institutions. None of the sponsors had been involved in the current study.
