# Supplementary material for: Efficacy and acceptability of different probiotic products plus laxatives for pediatric functional constipation: a network meta-analysis of randomized controlled trials
Source: Eur J Pediatr. 2024 May 29;183(8):3531–41. doi: 10.1007/s00431-024-05568-6 (PMC11263257; doi:10.1007/s00431-024-05568-6)
Supplement: Supplementary file 1 — Supplementary file1 (PPTX 745 kb) [file 431_2024_5568_MOESM1_ESM.pptx]

## Slide 1
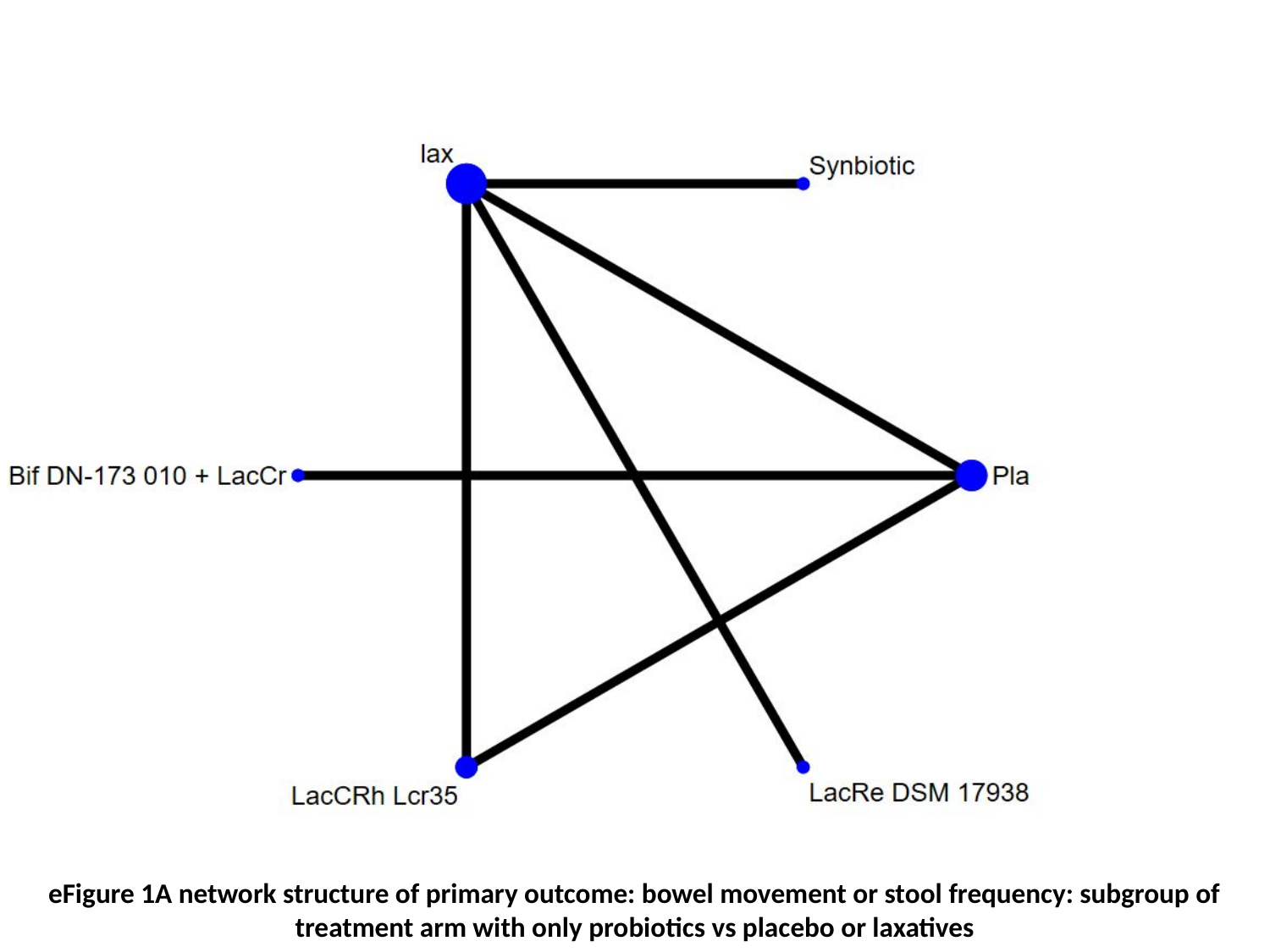

eFigure 1A network structure of primary outcome: bowel movement or stool frequency: subgroup of treatment arm with only probiotics vs placebo or laxatives

## Slide 2
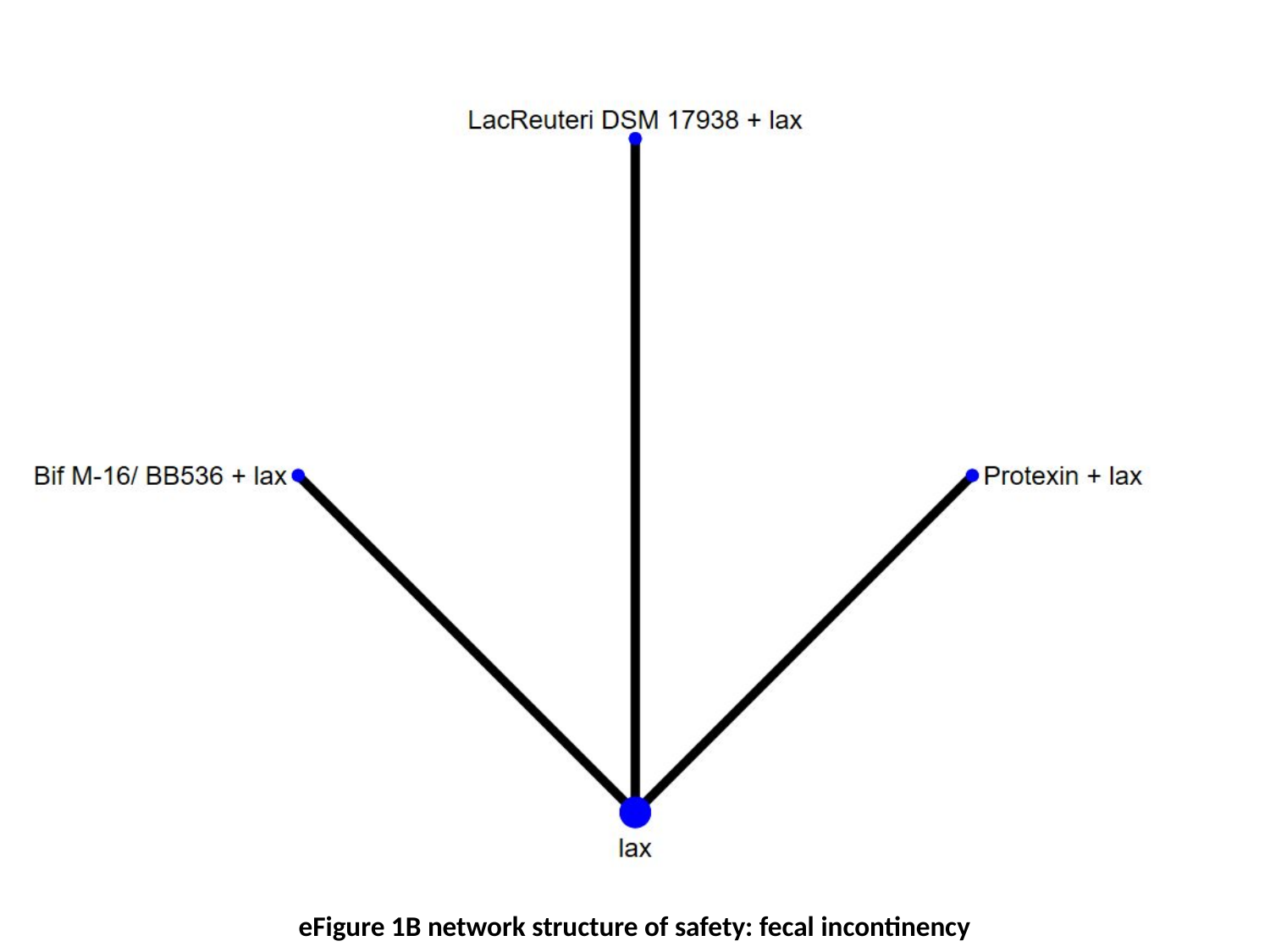

eFigure 1B network structure of safety: fecal incontinency

## Slide 3
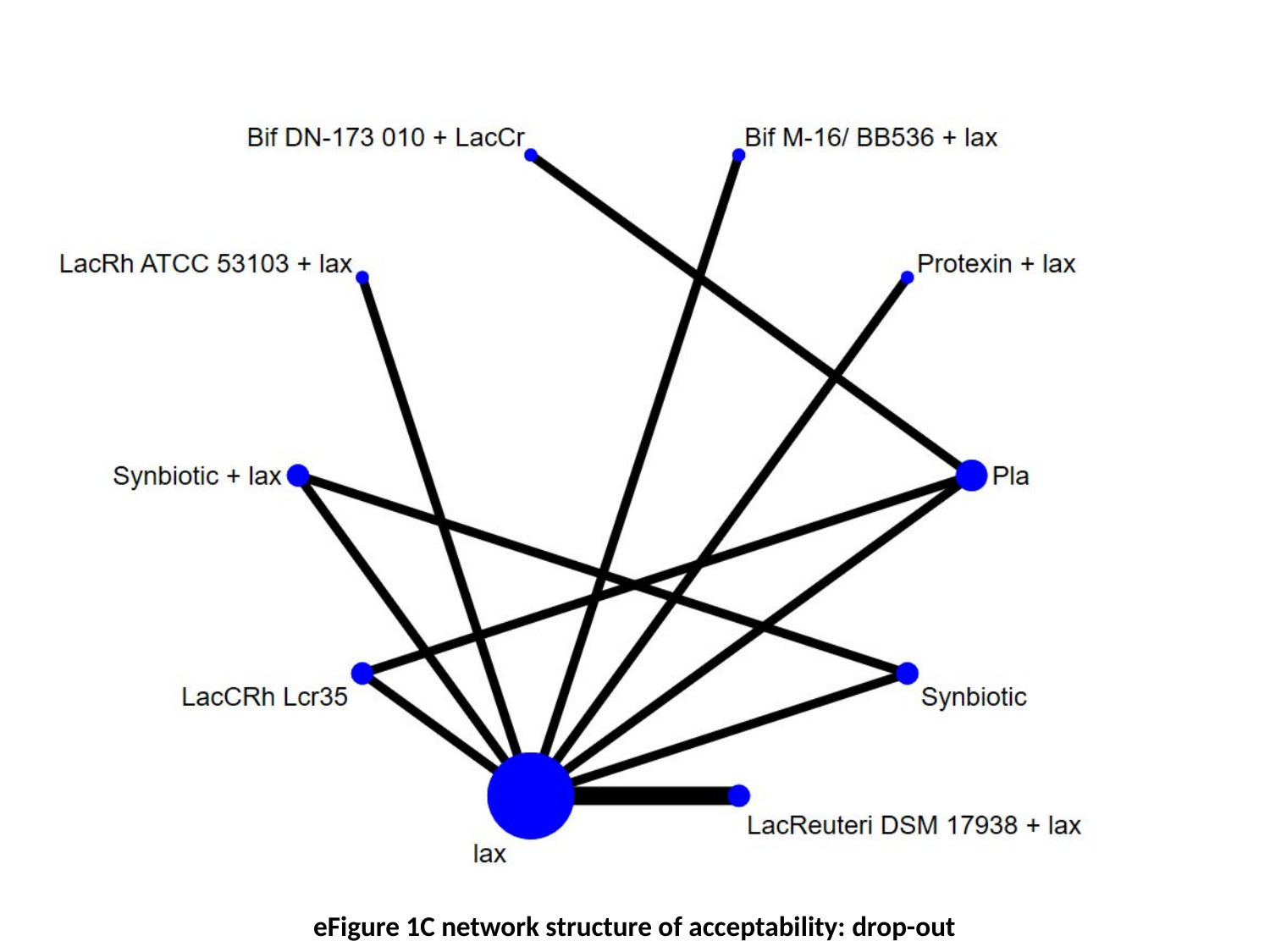

eFigure 1C network structure of acceptability: drop-out

## Slide 4
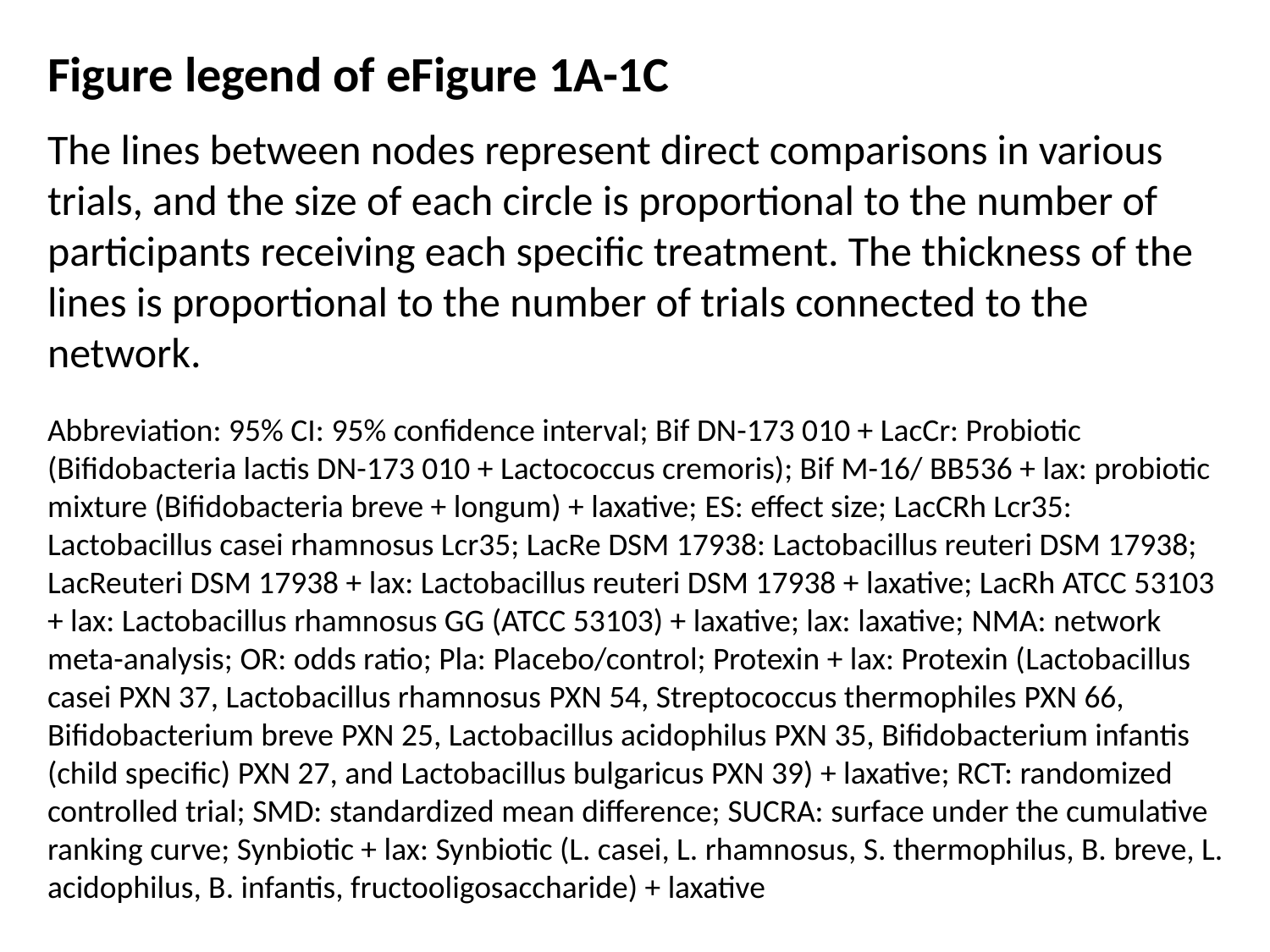

Figure legend of eFigure 1A-1C
The lines between nodes represent direct comparisons in various trials, and the size of each circle is proportional to the number of participants receiving each specific treatment. The thickness of the lines is proportional to the number of trials connected to the network.
Abbreviation: 95% CI: 95% confidence interval; Bif DN-173 010 + LacCr: Probiotic (Bifidobacteria lactis DN-173 010 + Lactococcus cremoris); Bif M-16/ BB536 + lax: probiotic mixture (Bifidobacteria breve + longum) + laxative; ES: effect size; LacCRh Lcr35: Lactobacillus casei rhamnosus Lcr35; LacRe DSM 17938: Lactobacillus reuteri DSM 17938; LacReuteri DSM 17938 + lax: Lactobacillus reuteri DSM 17938 + laxative; LacRh ATCC 53103 + lax: Lactobacillus rhamnosus GG (ATCC 53103) + laxative; lax: laxative; NMA: network meta-analysis; OR: odds ratio; Pla: Placebo/control; Protexin + lax: Protexin (Lactobacillus casei PXN 37, Lactobacillus rhamnosus PXN 54, Streptococcus thermophiles PXN 66, Bifidobacterium breve PXN 25, Lactobacillus acidophilus PXN 35, Bifidobacterium infantis (child specific) PXN 27, and Lactobacillus bulgaricus PXN 39) + laxative; RCT: randomized controlled trial; SMD: standardized mean difference; SUCRA: surface under the cumulative ranking curve; Synbiotic + lax: Synbiotic (L. casei, L. rhamnosus, S. thermophilus, B. breve, L. acidophilus, B. infantis, fructooligosaccharide) + laxative

## Slide 5
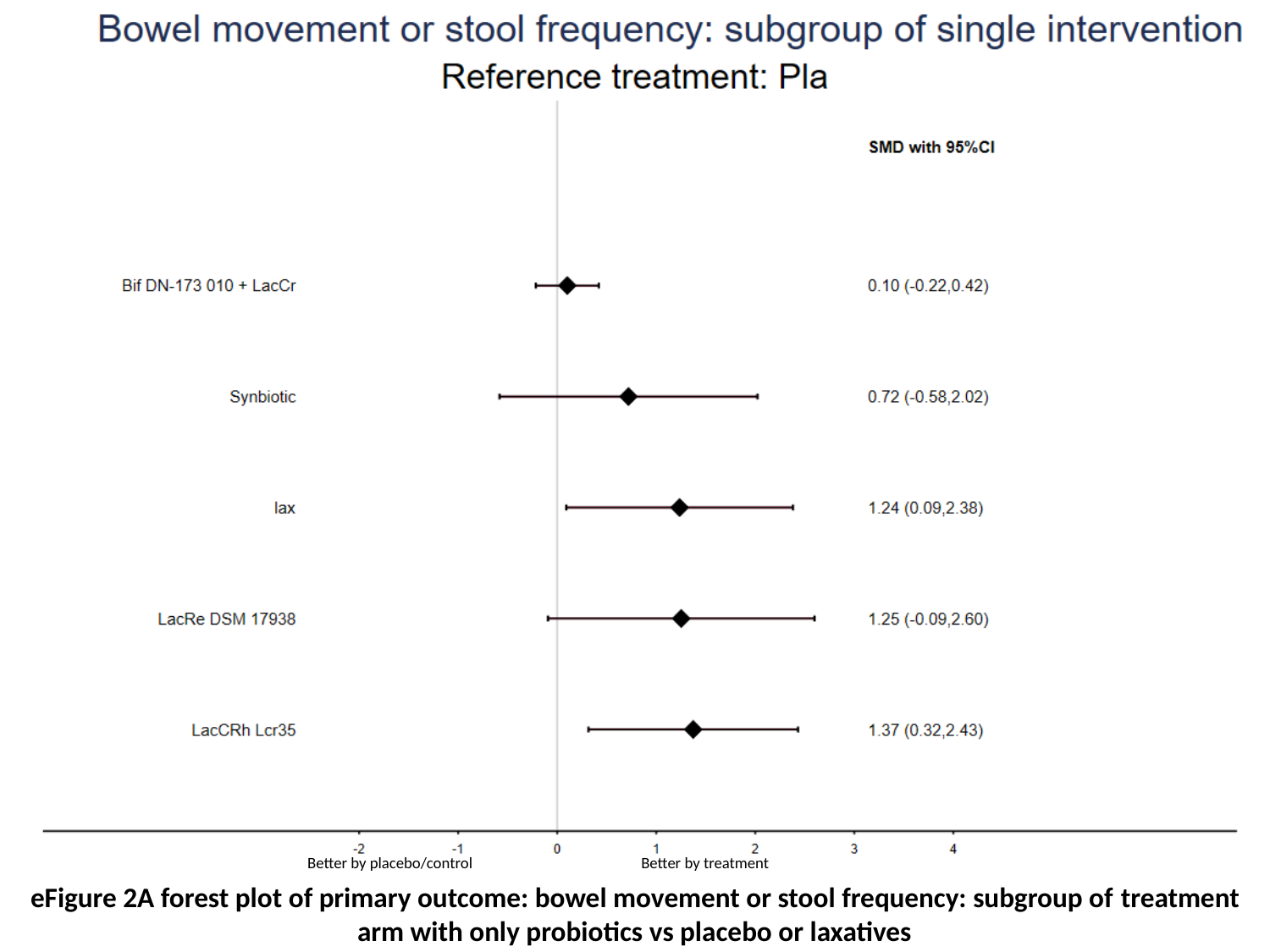

Better by placebo/control
Better by treatment
eFigure 2A forest plot of primary outcome: bowel movement or stool frequency: subgroup of treatment arm with only probiotics vs placebo or laxatives

## Slide 6
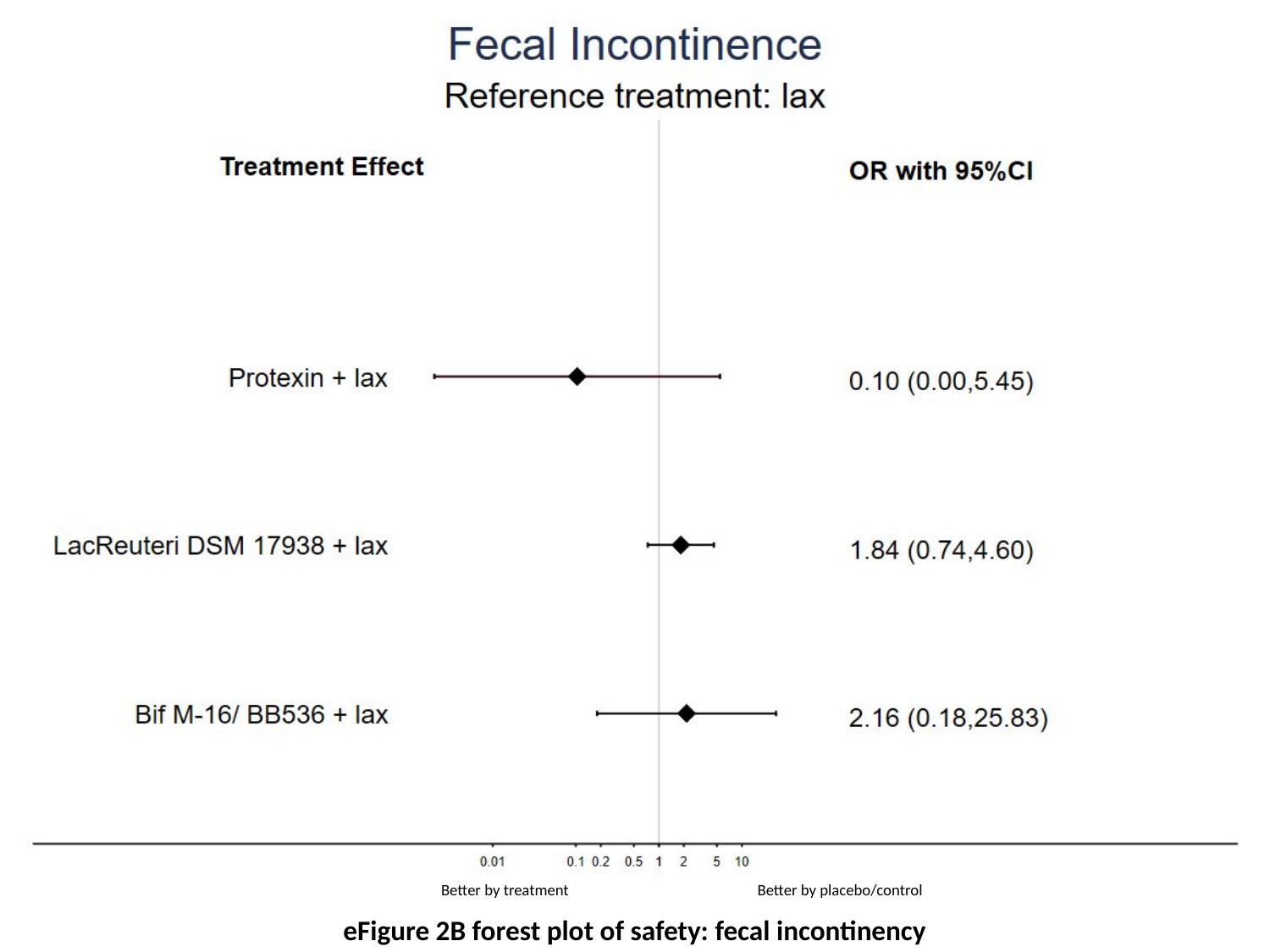

Better by treatment
Better by placebo/control
eFigure 2B forest plot of safety: fecal incontinency

## Slide 7
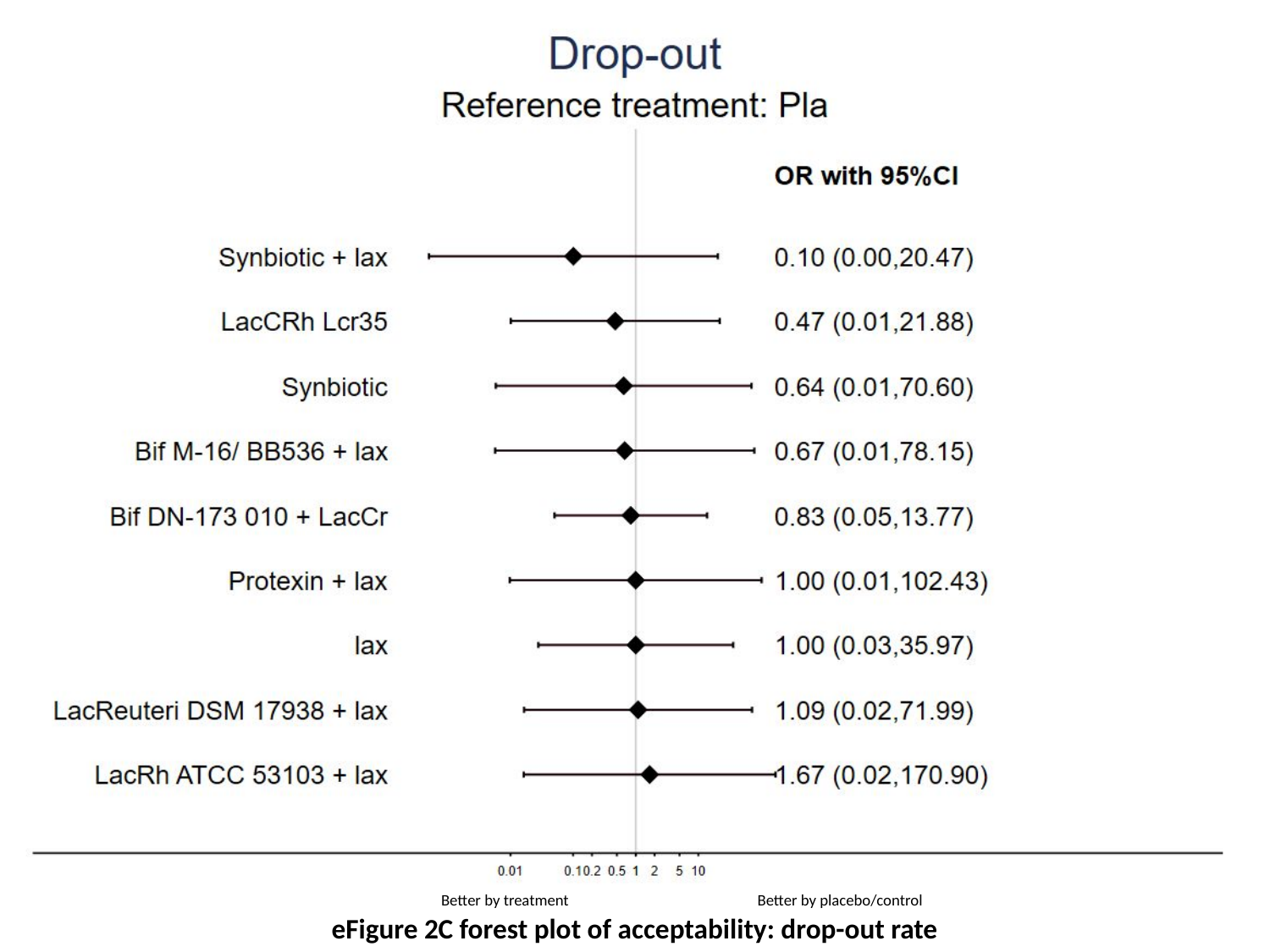

Better by treatment
Better by placebo/control
eFigure 2C forest plot of acceptability: drop-out rate

## Slide 8
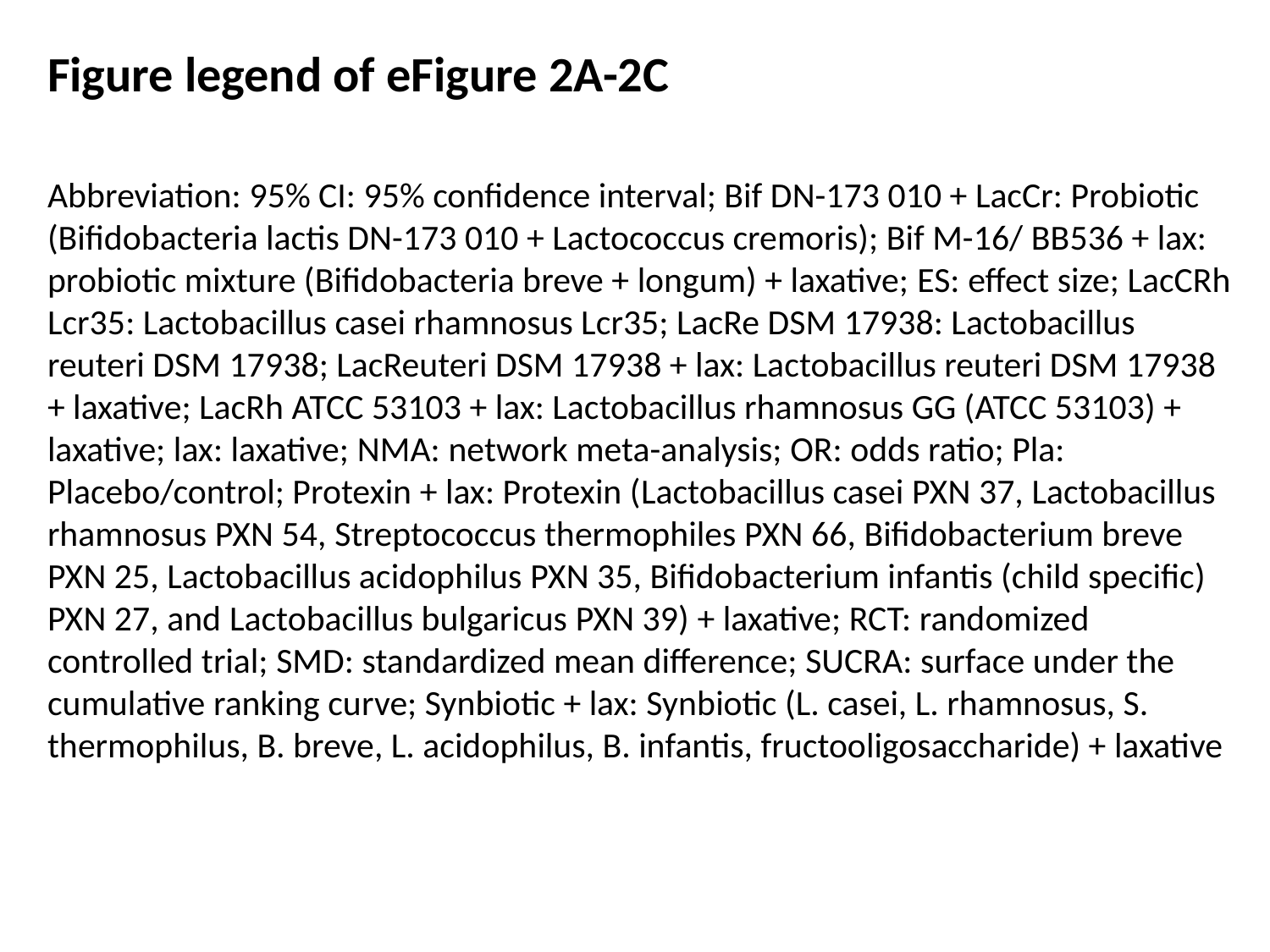

Figure legend of eFigure 2A-2C
Abbreviation: 95% CI: 95% confidence interval; Bif DN-173 010 + LacCr: Probiotic (Bifidobacteria lactis DN-173 010 + Lactococcus cremoris); Bif M-16/ BB536 + lax: probiotic mixture (Bifidobacteria breve + longum) + laxative; ES: effect size; LacCRh Lcr35: Lactobacillus casei rhamnosus Lcr35; LacRe DSM 17938: Lactobacillus reuteri DSM 17938; LacReuteri DSM 17938 + lax: Lactobacillus reuteri DSM 17938 + laxative; LacRh ATCC 53103 + lax: Lactobacillus rhamnosus GG (ATCC 53103) + laxative; lax: laxative; NMA: network meta-analysis; OR: odds ratio; Pla: Placebo/control; Protexin + lax: Protexin (Lactobacillus casei PXN 37, Lactobacillus rhamnosus PXN 54, Streptococcus thermophiles PXN 66, Bifidobacterium breve PXN 25, Lactobacillus acidophilus PXN 35, Bifidobacterium infantis (child specific) PXN 27, and Lactobacillus bulgaricus PXN 39) + laxative; RCT: randomized controlled trial; SMD: standardized mean difference; SUCRA: surface under the cumulative ranking curve; Synbiotic + lax: Synbiotic (L. casei, L. rhamnosus, S. thermophilus, B. breve, L. acidophilus, B. infantis, fructooligosaccharide) + laxative

## Slide 9
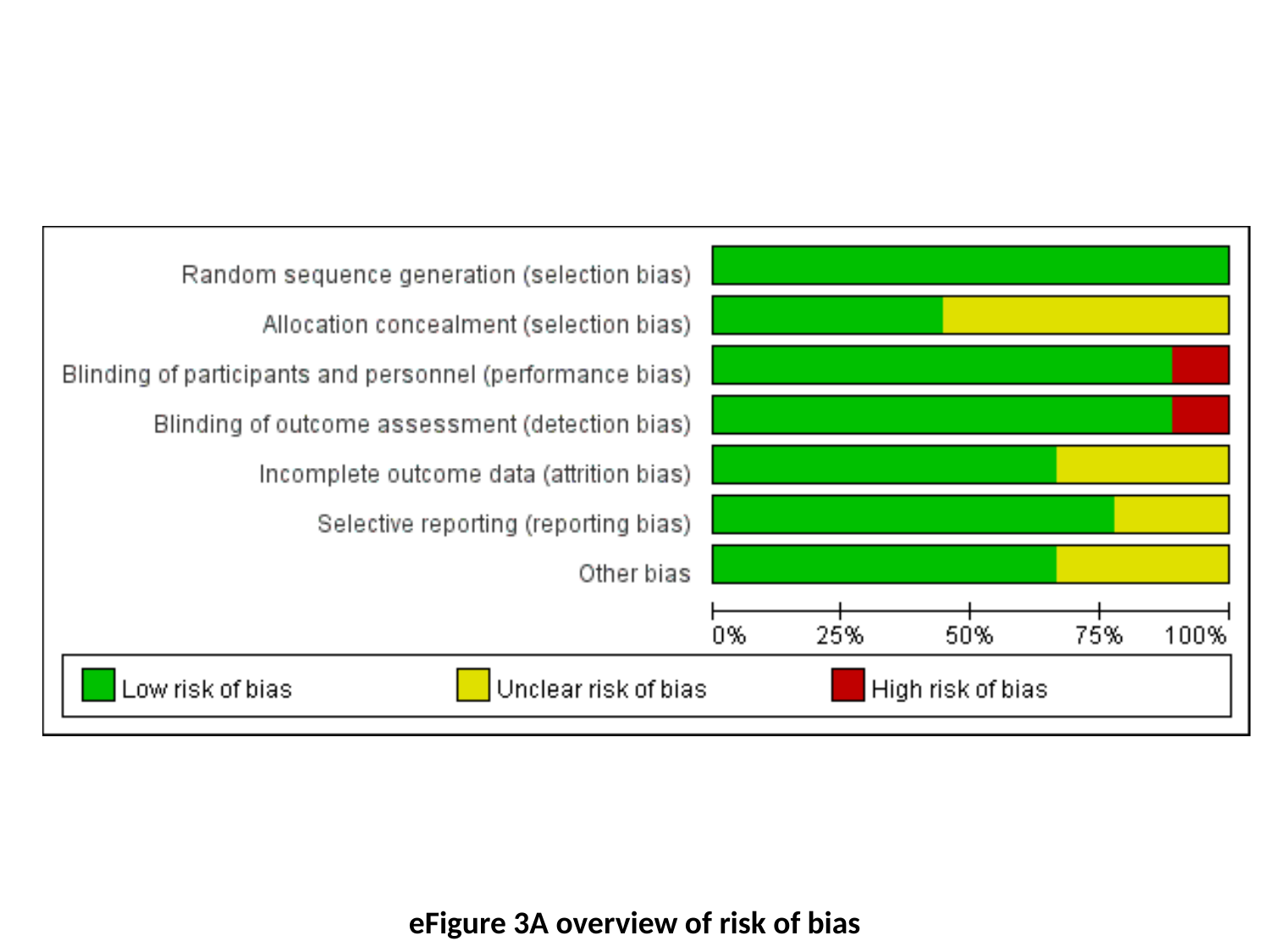

eFigure 3A overview of risk of bias

## Slide 10
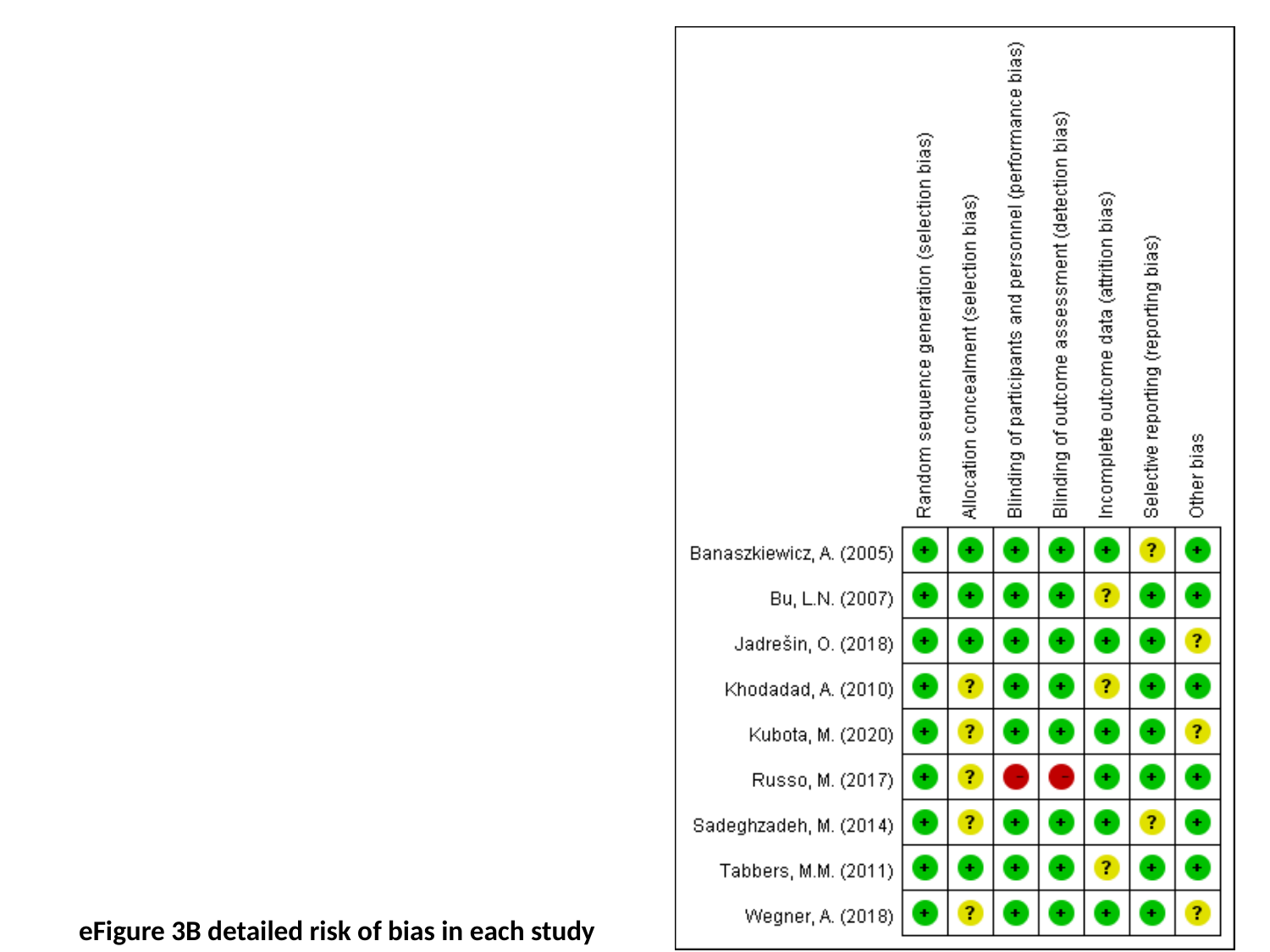

eFigure 3B detailed risk of bias in each study

## Slide 11
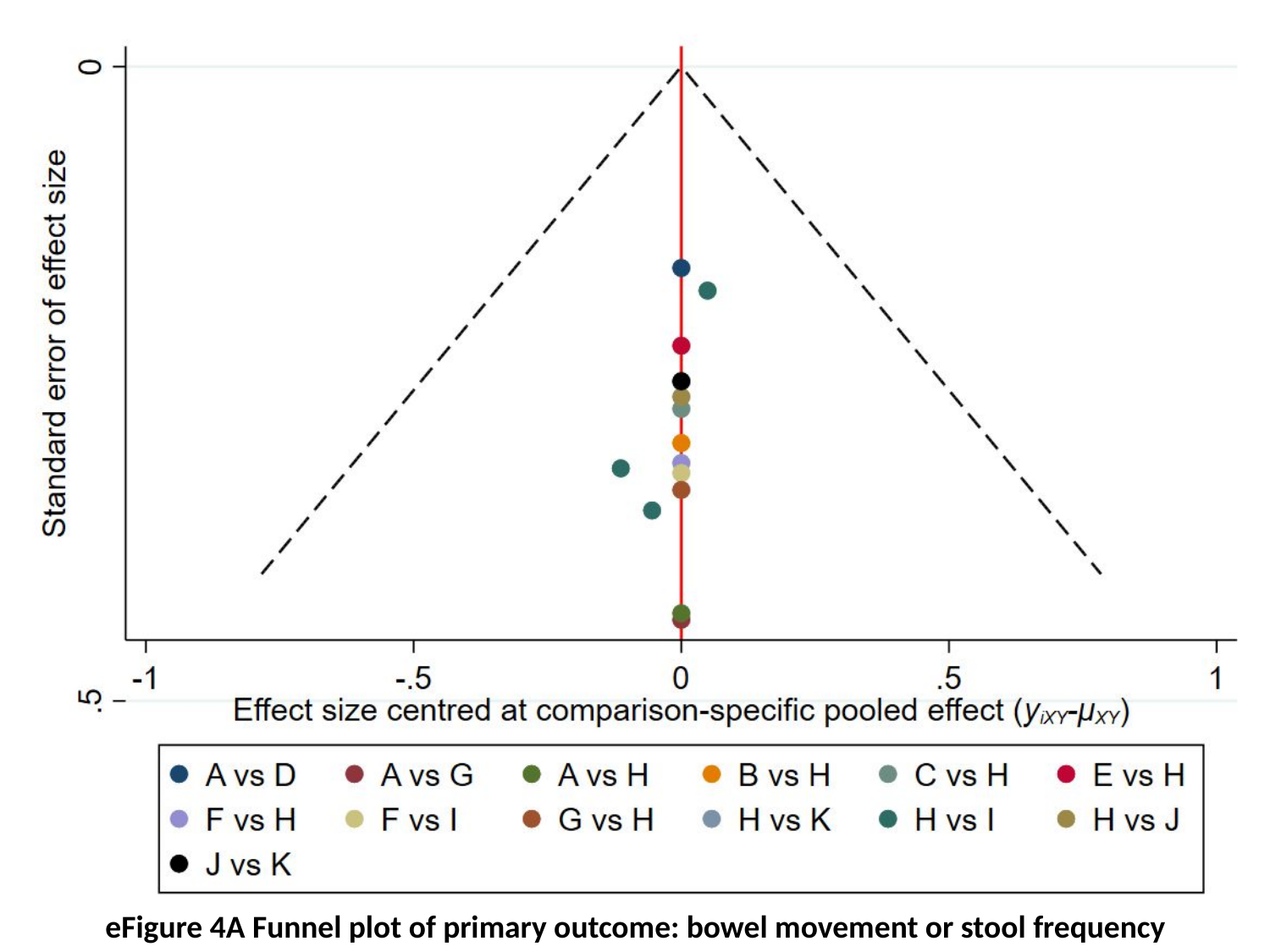

eFigure 4A Funnel plot of primary outcome: bowel movement or stool frequency

## Slide 12
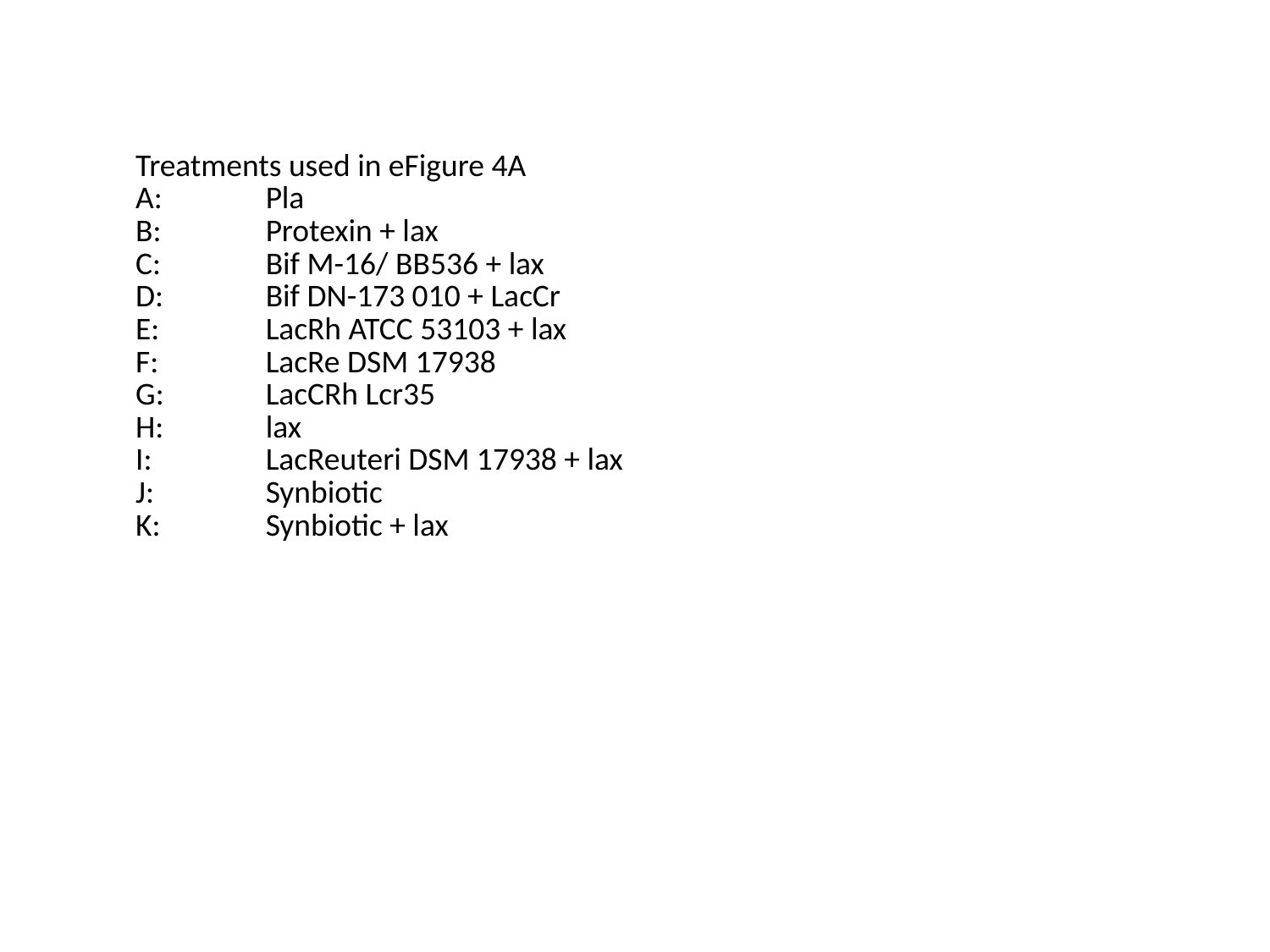

| Treatments used in eFigure 4A | |
| --- | --- |
| A: | Pla |
| B: | Protexin + lax |
| C: | Bif M-16/ BB536 + lax |
| D: | Bif DN-173 010 + LacCr |
| E: | LacRh ATCC 53103 + lax |
| F: | LacRe DSM 17938 |
| G: | LacCRh Lcr35 |
| H: | lax |
| I: | LacReuteri DSM 17938 + lax |
| J: | Synbiotic |
| K: | Synbiotic + lax |

## Slide 13
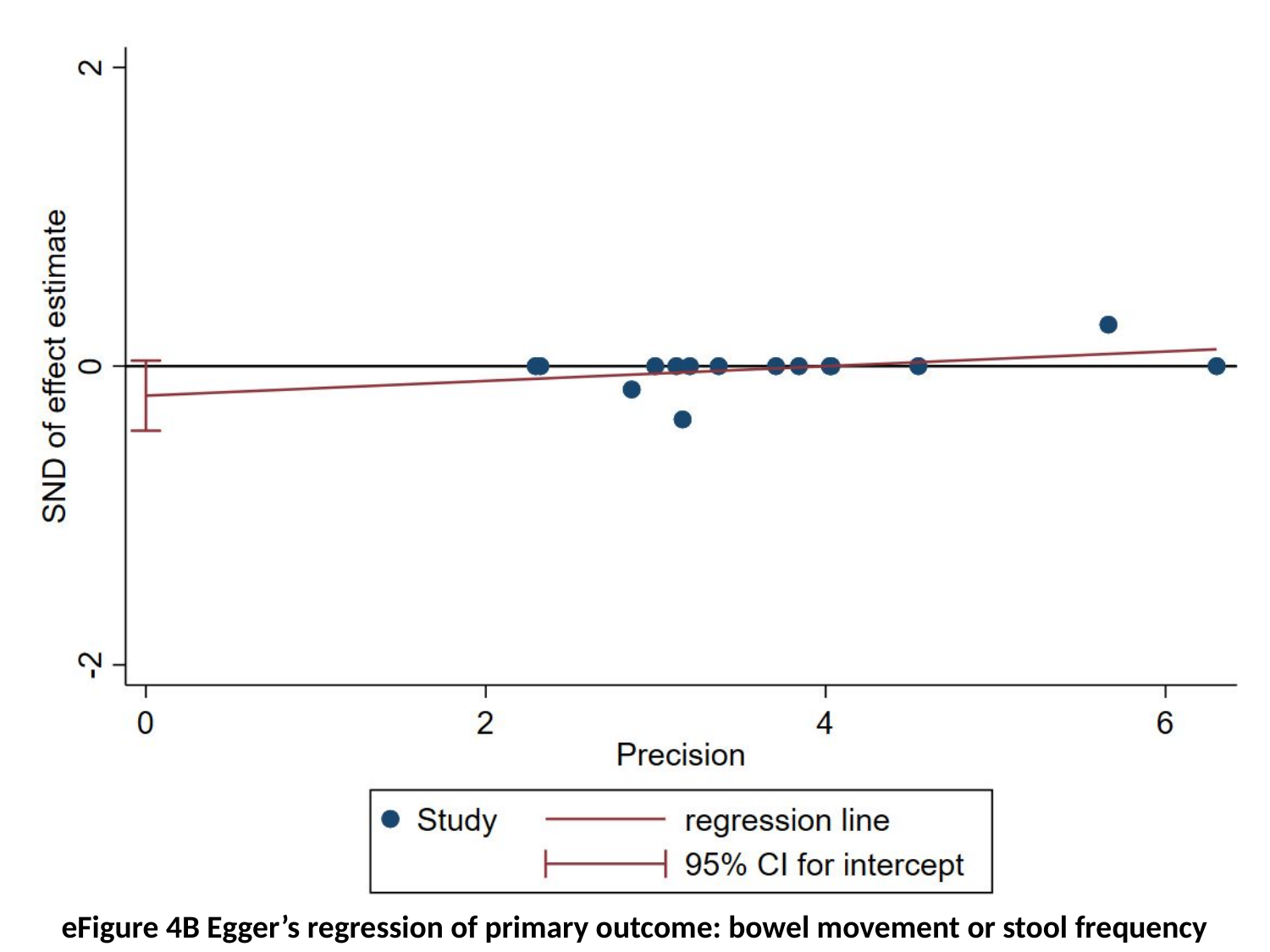

eFigure 4B Egger’s regression of primary outcome: bowel movement or stool frequency

## Slide 14
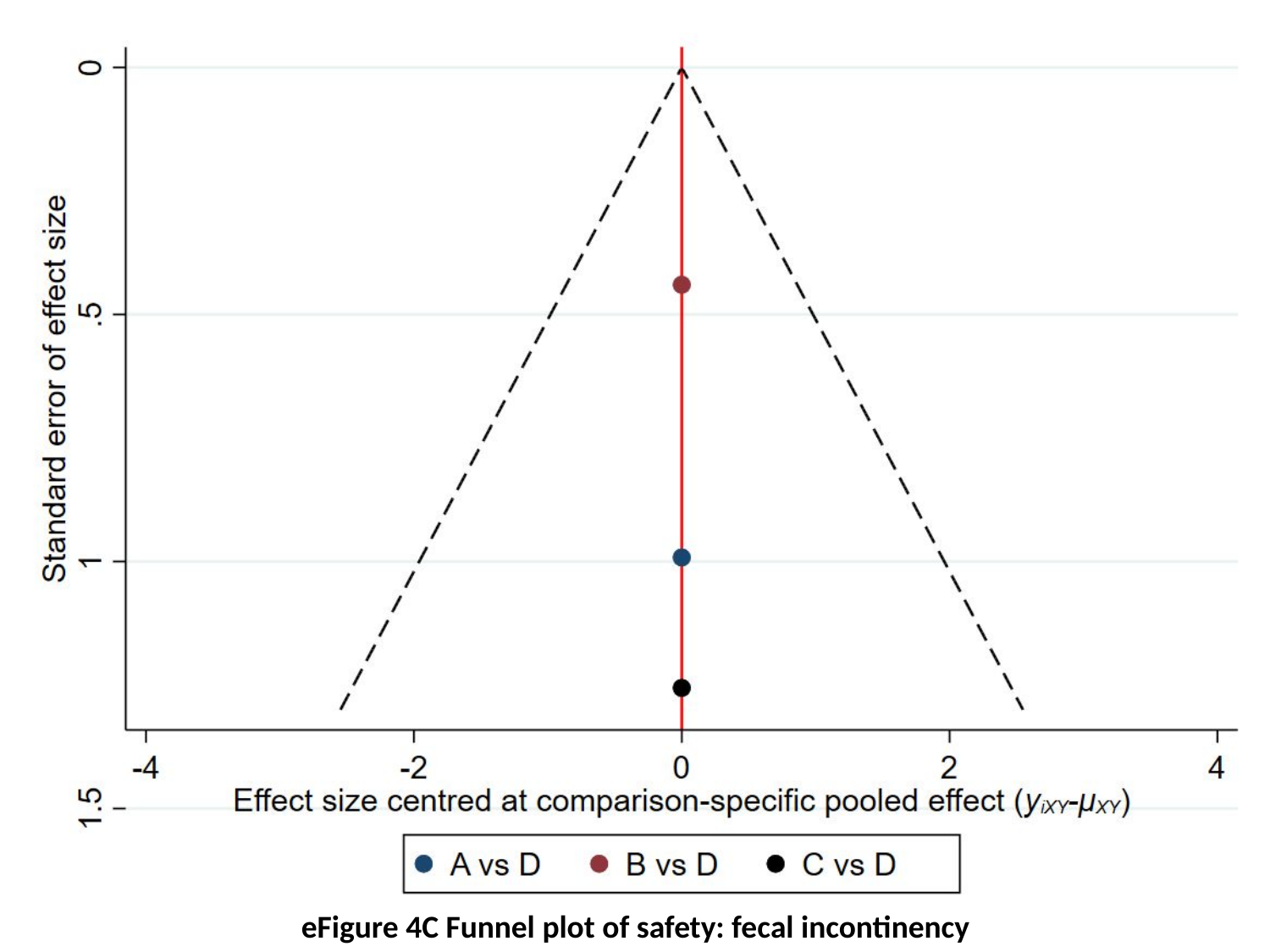

eFigure 4C Funnel plot of safety: fecal incontinency

## Slide 15
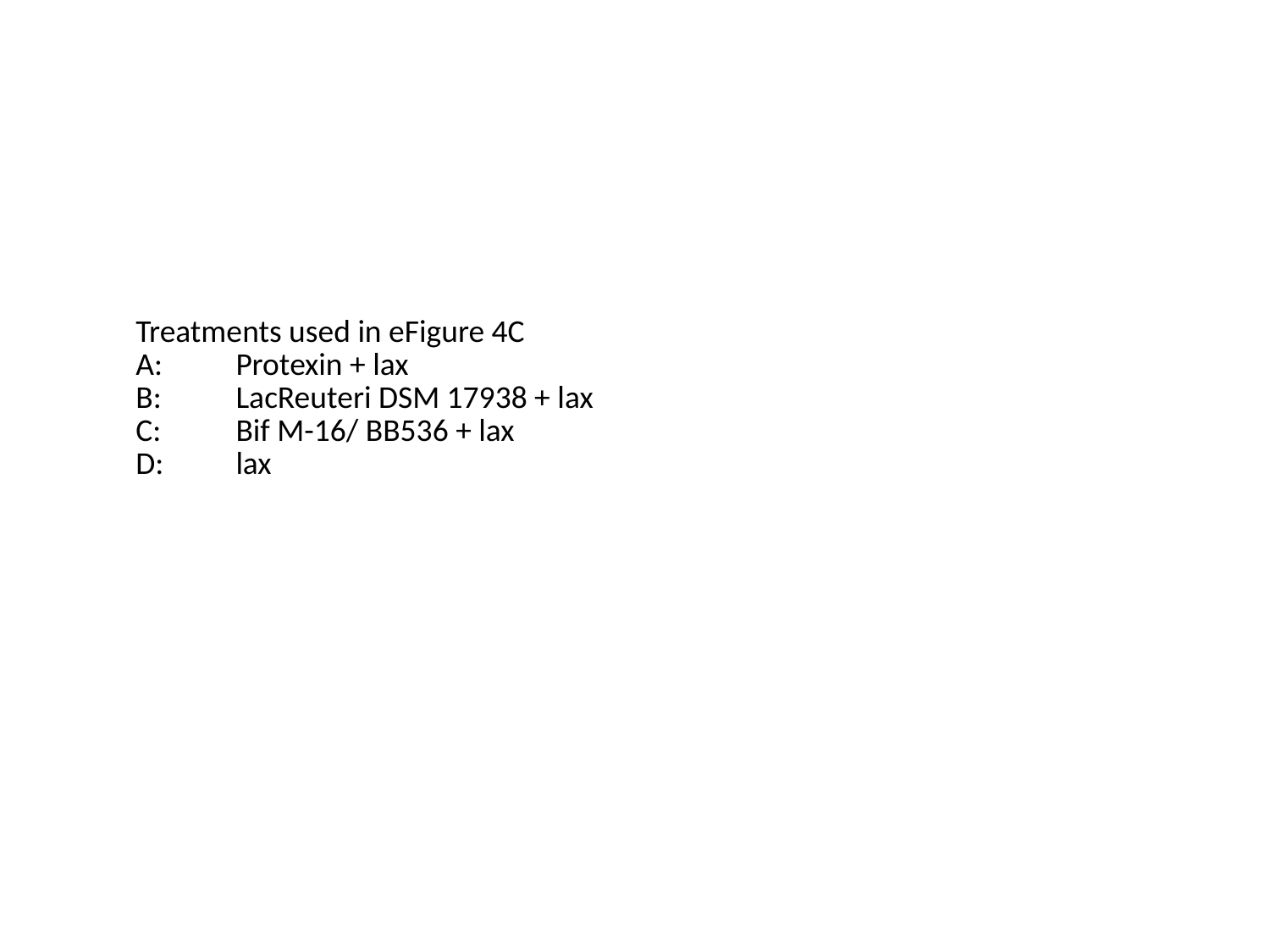

| Treatments used in eFigure 4C | |
| --- | --- |
| A: | Protexin + lax |
| B: | LacReuteri DSM 17938 + lax |
| C: | Bif M-16/ BB536 + lax |
| D: | lax |

## Slide 16
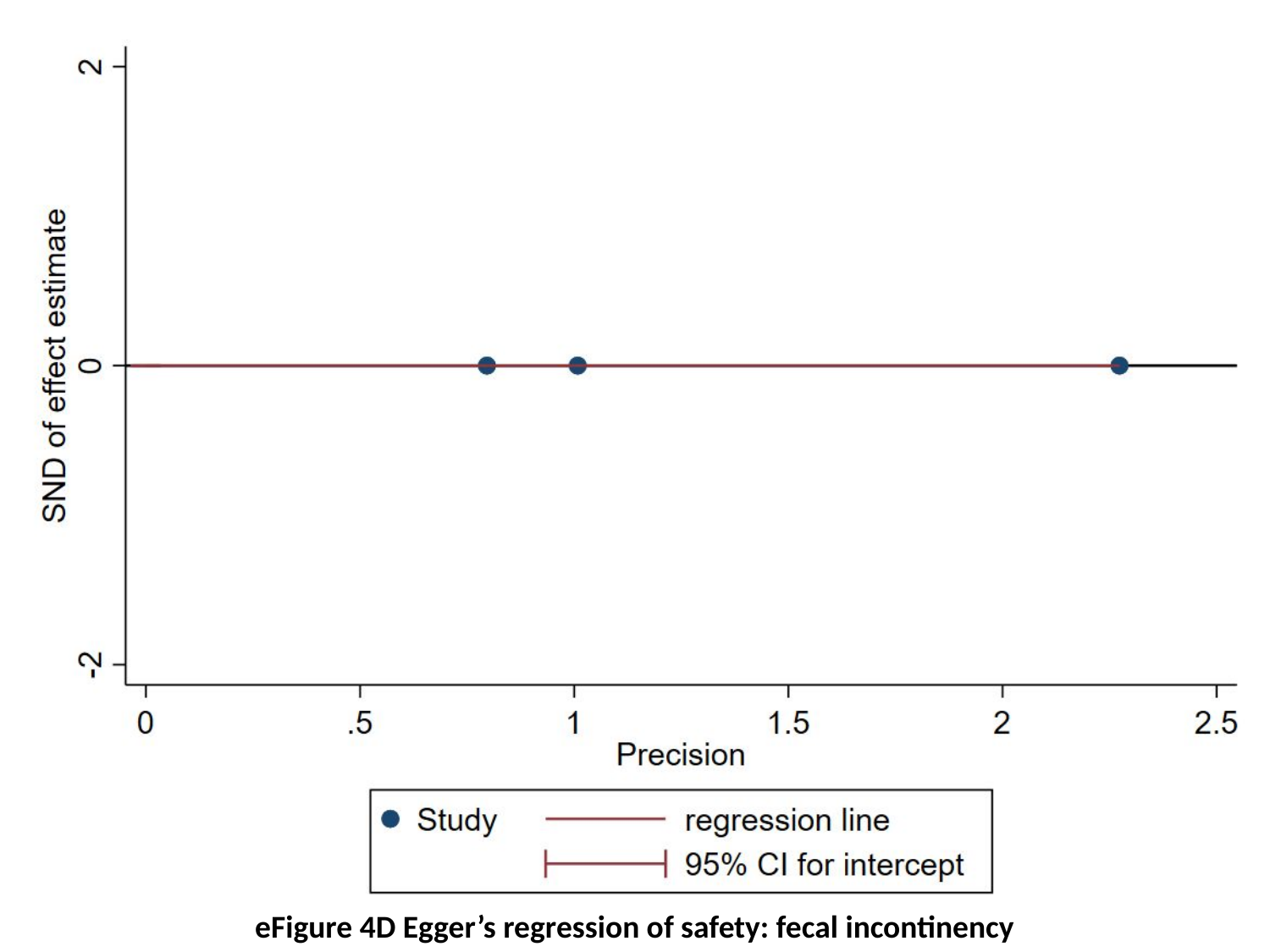

eFigure 4D Egger’s regression of safety: fecal incontinency

## Slide 17
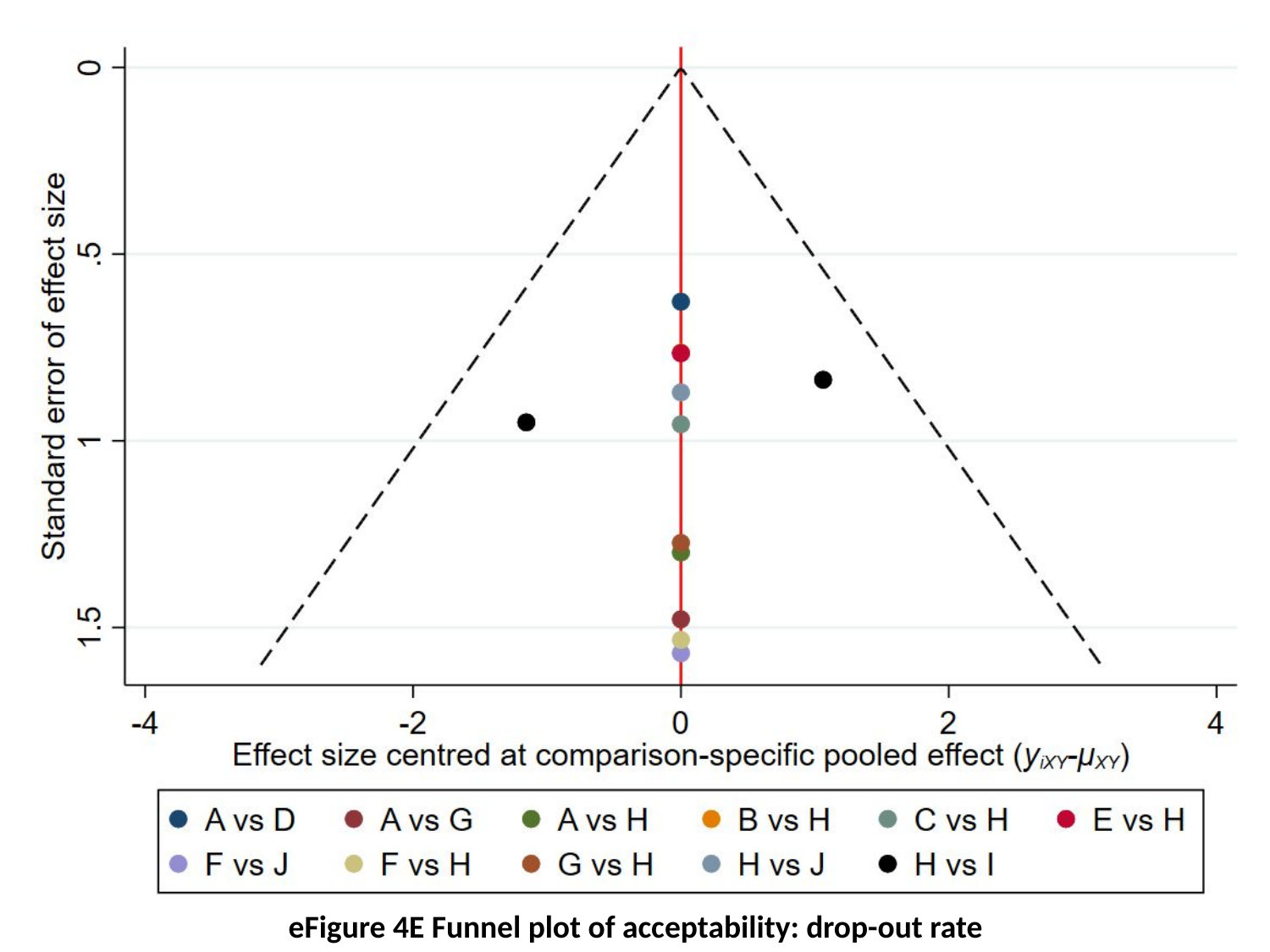

eFigure 4E Funnel plot of acceptability: drop-out rate

## Slide 18
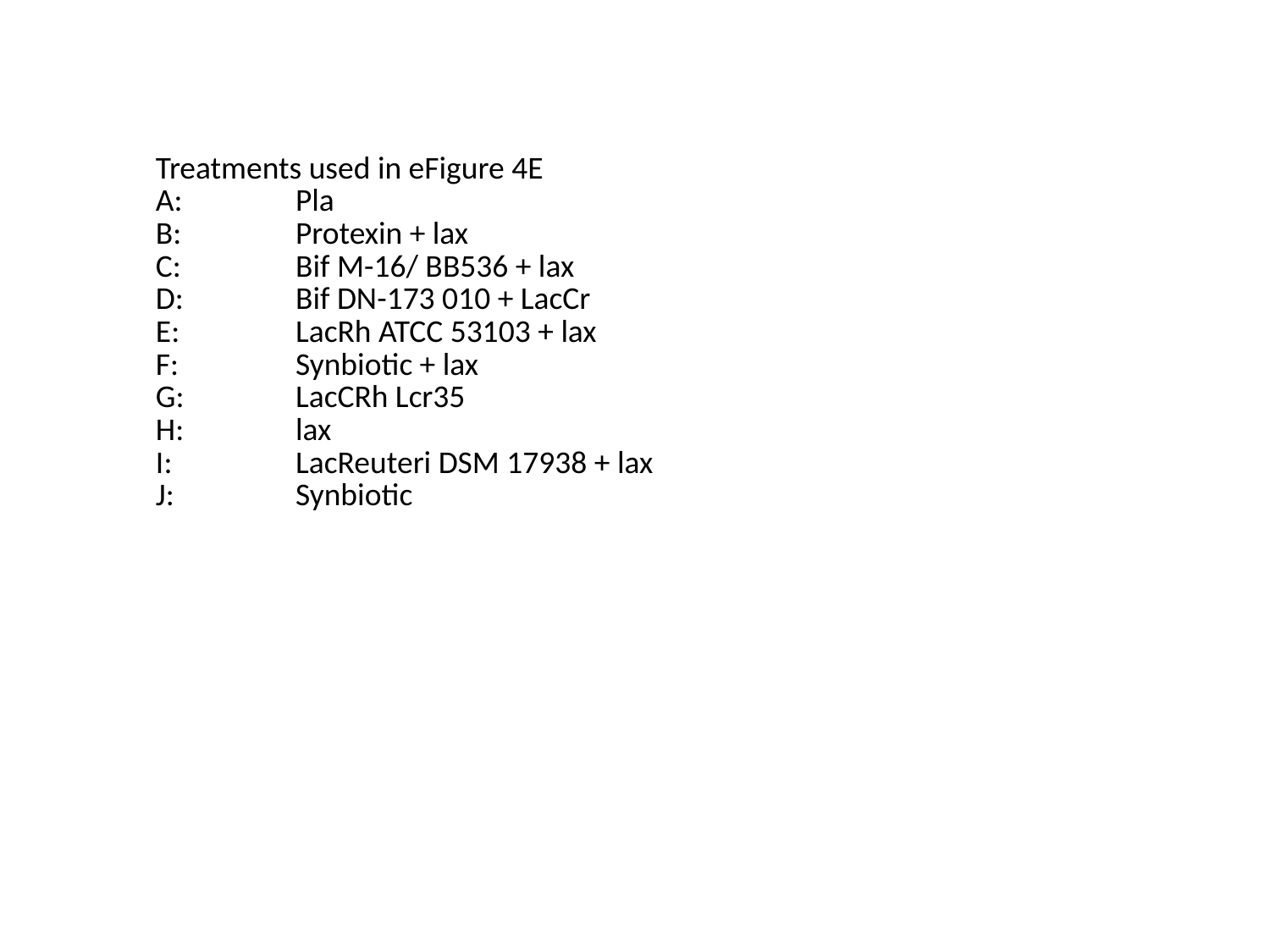

| Treatments used in eFigure 4E | |
| --- | --- |
| A: | Pla |
| B: | Protexin + lax |
| C: | Bif M-16/ BB536 + lax |
| D: | Bif DN-173 010 + LacCr |
| E: | LacRh ATCC 53103 + lax |
| F: | Synbiotic + lax |
| G: | LacCRh Lcr35 |
| H: | lax |
| I: | LacReuteri DSM 17938 + lax |
| J: | Synbiotic |

## Slide 19
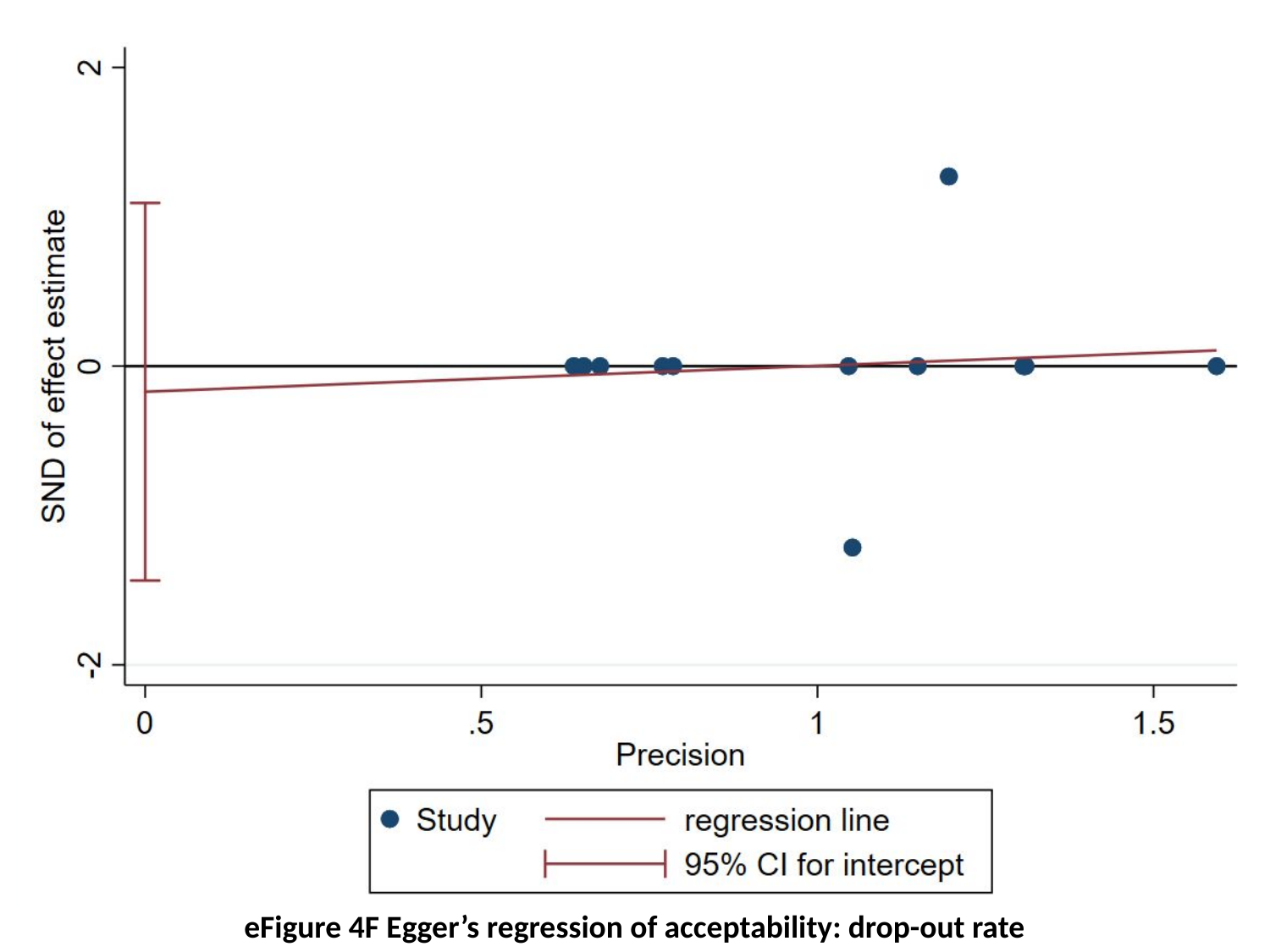

eFigure 4F Egger’s regression of acceptability: drop-out rate

## Slide 20
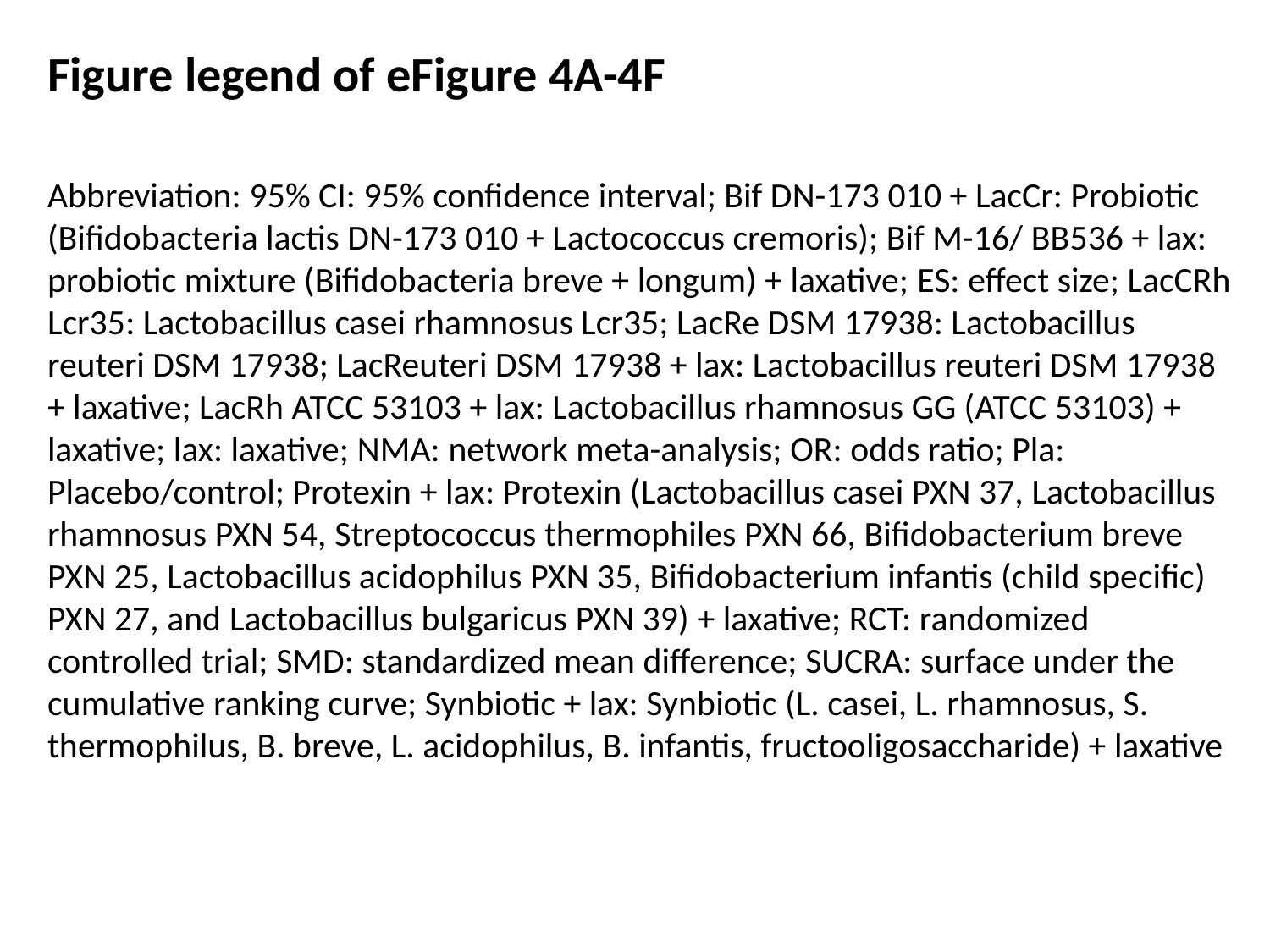

Figure legend of eFigure 4A-4F
Abbreviation: 95% CI: 95% confidence interval; Bif DN-173 010 + LacCr: Probiotic (Bifidobacteria lactis DN-173 010 + Lactococcus cremoris); Bif M-16/ BB536 + lax: probiotic mixture (Bifidobacteria breve + longum) + laxative; ES: effect size; LacCRh Lcr35: Lactobacillus casei rhamnosus Lcr35; LacRe DSM 17938: Lactobacillus reuteri DSM 17938; LacReuteri DSM 17938 + lax: Lactobacillus reuteri DSM 17938 + laxative; LacRh ATCC 53103 + lax: Lactobacillus rhamnosus GG (ATCC 53103) + laxative; lax: laxative; NMA: network meta-analysis; OR: odds ratio; Pla: Placebo/control; Protexin + lax: Protexin (Lactobacillus casei PXN 37, Lactobacillus rhamnosus PXN 54, Streptococcus thermophiles PXN 66, Bifidobacterium breve PXN 25, Lactobacillus acidophilus PXN 35, Bifidobacterium infantis (child specific) PXN 27, and Lactobacillus bulgaricus PXN 39) + laxative; RCT: randomized controlled trial; SMD: standardized mean difference; SUCRA: surface under the cumulative ranking curve; Synbiotic + lax: Synbiotic (L. casei, L. rhamnosus, S. thermophilus, B. breve, L. acidophilus, B. infantis, fructooligosaccharide) + laxative
