# Supplementary material for: Efficacy and acceptability of different probiotic products plus laxatives for pediatric functional constipation: a network meta-analysis of randomized controlled trials
Source: Eur J Pediatr. 2024 May 29;183(8):3531–41. doi: 10.1007/s00431-024-05568-6 (PMC11263257; doi:10.1007/s00431-024-05568-6)
Supplement: Supplementary file 2 — Supplementary file2 (DOCX 161 kb) [file 431_2024_5568_MOESM2_ESM.docx]

**eTable 1:** PRISMA 2020 checklist of current network meta-analysis

| **Section and Topic** | **Item #** | **Checklist item** | **Page where item is reported** |
| --- | --- | --- | --- |
| **TITLE** | | |  |
| Title | 1 | Identify the report as a systematic review. | 1 |
| **ABSTRACT** | | |  |
| Abstract | 2 | See the PRISMA 2020 for Abstracts checklist. | 6-7 |
| **INTRODUCTION** | | |  |
| Rationale | 3 | Describe the rationale for the review in the context of existing knowledge. | 8-9 |
| Objectives | 4 | Provide an explicit statement of the objective(s) or question(s) the review addresses. | 8-9 |
| **METHODS** | | |  |
| Eligibility criteria | 5 | Specify the inclusion and exclusion criteria for the review and how studies were grouped for the syntheses. | 10-11 |
| Information sources | 6 | Specify all databases, registers, websites, organisations, reference lists and other sources searched or consulted to identify studies. Specify the date when each source was last searched or consulted. | 10-11 |
| Search strategy | 7 | Present the full search strategies for all databases, registers and websites, including any filters and limits used. | 10-11 |
| Selection process | 8 | Specify the methods used to decide whether a study met the inclusion criteria of the review, including how many reviewers screened each record and each report retrieved, whether they worked independently, and if applicable, details of automation tools used in the process. | 10-11 |
| Data collection process | 9 | Specify the methods used to collect data from reports, including how many reviewers collected data from each report, whether they worked independently, any processes for obtaining or confirming data from study investigators, and if applicable, details of automation tools used in the process. | 10-11 |
| Data items | 10a | List and define all outcomes for which data were sought. Specify whether all results that were compatible with each outcome domain in each study were sought (e.g. for all measures, time points, analyses), and if not, the methods used to decide which results to collect. | 10-11 |
|  | 10b | List and define all other variables for which data were sought (e.g. participant and intervention characteristics, funding sources). Describe any assumptions made about any missing or unclear information. | 10-11 |
| Study risk of bias assessment | 11 | Specify the methods used to assess risk of bias in the included studies, including details of the tool(s) used, how many reviewers assessed each study and whether they worked independently, and if applicable, details of automation tools used in the process. | 10-11 |
| Effect measures | 12 | Specify for each outcome the effect measure(s) (e.g. risk ratio, mean difference) used in the synthesis or presentation of results. | 10-11 |
| Synthesis methods | 13a | Describe the processes used to decide which studies were eligible for each synthesis (e.g. tabulating the study intervention characteristics and comparing against the planned groups for each synthesis (item #5)). | 10-11 |
|  | 13b | Describe any methods required to prepare the data for presentation or synthesis, such as handling of missing summary statistics, or data conversions. | 10-11 |
|  | 13c | Describe any methods used to tabulate or visually display results of individual studies and syntheses. | 11-12 |
|  | 13d | Describe any methods used to synthesize results and provide a rationale for the choice(s). If meta-analysis was performed, describe the model(s), method(s) to identify the presence and extent of statistical heterogeneity, and software package(s) used. | 11-12 |
|  | 13e | Describe any methods used to explore possible causes of heterogeneity among study results (e.g. subgroup analysis, meta-regression). | 11-12 |
|  | 13f | Describe any sensitivity analyses conducted to assess robustness of the synthesized results. | 11-12 |
| Reporting bias assessment | 14 | Describe any methods used to assess risk of bias due to missing results in a synthesis (arising from reporting biases). | 11-12 |
| Certainty assessment | 15 | Describe any methods used to assess certainty (or confidence) in the body of evidence for an outcome. | 11-12 |
| **RESULTS** | | |  |
| Study selection | 16a | Describe the results of the search and selection process, from the number of records identified in the search to the number of studies included in the review, ideally using a flow diagram. | 13-14, Fig 1 |
|  | 16b | Cite studies that might appear to meet the inclusion criteria, but which were excluded, and explain why they were excluded. | 13-14, eTab 2 |
| Study characteristics | 17 | Cite each included study and present its characteristics. | 13-14, eTab 3 |
| Risk of bias in studies | 18 | Present assessments of risk of bias for each included study. | 13-14, eFig 3 |
| Results of individual studies | 19 | For all outcomes, present, for each study: (a) summary statistics for each group (where appropriate) and (b) an effect estimate and its precision (e.g. confidence/credible interval), ideally using structured tables or plots. | 13-14, eTab 3 |
| Results of syntheses | 20a | For each synthesis, briefly summarise the characteristics and risk of bias among contributing studies. | 13-14, eFig 3 |
|  | 20b | Present results of all statistical syntheses conducted. If meta-analysis was done, present for each the summary estimate and its precision (e.g. confidence/credible interval) and measures of statistical heterogeneity. If comparing groups, describe the direction of the effect. | 13-14, Fig 3, eFig 2 |
|  | 20c | Present results of all investigations of possible causes of heterogeneity among study results. | 13-14, eTab 6-7 |
|  | 20d | Present results of all sensitivity analyses conducted to assess the robustness of the synthesized results. | 13-14 |
| Reporting biases | 21 | Present assessments of risk of bias due to missing results (arising from reporting biases) for each synthesis assessed. | 13-14, eFig 3 |
| Certainty of evidence | 22 | Present assessments of certainty (or confidence) in the body of evidence for each outcome assessed. | 13-14, eTab 6-7 |
| **DISCUSSION** | | |  |
| Discussion | 23a | Provide a general interpretation of the results in the context of other evidence. | 15-16 |
|  | 23b | Discuss any limitations of the evidence included in the review. | 17-18 |
|  | 23c | Discuss any limitations of the review processes used. | 17-18 |
|  | 23d | Discuss implications of the results for practice, policy, and future research. | 19 |
| **OTHER INFORMATION** | | |  |
| Registration and protocol | 24a | Provide registration information for the review, including register name and registration number, or state that the review was not registered. | 7 |
|  | 24b | Indicate where the review protocol can be accessed, or state that a protocol was not prepared. | 7 |
|  | 24c | Describe and explain any amendments to information provided at registration or in the protocol. | 7 |
| Support | 25 | Describe sources of financial or non-financial support for the review, and the role of the funders or sponsors in the review. | 20 |
| Competing interests | 26 | Declare any competing interests of review authors. | 20 |
| Availability of data, code and other materials | 27 | Report which of the following are publicly available and where they can be found: template data collection forms; data extracted from included studies; data used for all analyses; analytic code; any other materials used in the review. | 20 |

The current checklist followed the latest PRISMA 2020 guideline [1].

**eTable 2: keyword used in each database and search result**

| Database | Keyword | Filter | Date | Result |
| --- | --- | --- | --- | --- |
| PubMed | (functional constipation OR functional constipation[MeSH Terms] OR constipation OR constipation[MeSH Terms]) AND (Probiotics[MeSH Terms] OR Probiotics) AND (paediatric OR pediatric OR child OR infant OR baby OR juvenile OR kid) AND (random OR randomized OR randomised) | NA | 2021/12/16 | 77 |
| Embase | (functional constipation OR constipation) AND (Probiotics) AND (paediatric OR pediatric OR child OR infant OR baby OR juvenile OR kid) AND (random OR randomized OR randomised) | NA | 2021/12/16 | 188 |
| ClinicalKey | (probiotics) AND (functional constipation) AND (child OR pediatric) | NA | 2021/12/16 | 179 |
| Cochrane CENTRAL | (functional constipation OR constipation) AND (Probiotics) AND (paediatric OR pediatric OR child OR infant OR baby OR juvenile OR kid) AND (random OR randomized OR randomised) | NA | 2021/12/16 | 96 |
| ProQuest | (probiotics) AND (functional constipation) AND (child OR pediatric) | NA | 2021/12/16 | 770 |
| ScienceDirect | (probiotics) AND (functional constipation) AND (child OR pediatric) | research article | 2021/12/16 | 190 |
| Web of Science | (functional constipation OR constipation) AND (Probiotics) AND (paediatric OR pediatric OR child OR infant OR baby OR juvenile OR kid) AND (random OR randomized OR randomised) | NA | 2021/12/16 | 78 |
| ClinicalTrials.gov | (probiotics) AND (functional constipation) AND (child OR pediatric) | NA | 2021/12/16 | 2 |

Abbreviation: NA: not applied

**eTable 3: Excluded studies and reason**

| Reason | Number | Reference |
| --- | --- | --- |
| Also consisted of diarrhea but not constipation simply | 3 | [2-4] |
| Chemotherapy-induced gastrointestinal problem but not functional constipation | 1 | [5] |
| Comorbid with other neurologic disease, neurometabolic disease, or neuropsychiatric disease, which would violate the similarity hypothesis | 4 | [6-9] |
| Could not form the network structure (both arm had yogurt with B. longum strain, which could not fit in other treatment arms) | 1 | [10] |
| Insufficient data available | 1 | [11] |
| Meta-analysis | 13 | [12-24] |
| Not associated with probiotics in functional constipation | 10 | [25-34] |
| Not pediatric subjects | 5 | [35-39] |
| Not randomized controlled trials | 3 | [40-42] |
| Prevention but not treatment | 1 | [43] |
| Protocol of clinical study or meta-analysis | 1 | [44; 45] |
| Review article | 11 | [46-56] |
| Unusually huge placebo effect (70%) | 1 | [57] |

**eTable 4: Characteristics of the included studies**

| Study name | Baseline constipation severity | Diagnosis of constipation | Comparison | Subject | mean age | female proportion | Tx duration (week) | Study duration (week)^%^ | Country |
| --- | --- | --- | --- | --- | --- | --- | --- | --- | --- |
| [58] | <= 2 defecations per week | Rome IV Criteria | L reuteri DSM 17938 L reuteri DSM 17938 + MgO MgO | 20 19 21 | 2.7±1.3 3.4±1.5 2.9±1.3 | 45.0 47.4 42.9 | 4 | 4 | Japan |
| [59] | not mentioned | Rome III Criteria | L reuteri DSM 17938 + lactulose Placebo + lactulose | 18 15 | 4.4 4.7 | 66.7 73.3 | 12 | 16 | Croatia |
| [60] | < 3 bowel movement per week at least 8 weeks | Rome III Criteria | L reuteri DSM 17938 + macrogol therapy macrogol therapy + placebo | 65 64 | 4.7±1.3 4.7±1.3 | 44.6 43.8 | 8 | 8 | Poland |
| [61] | < 3 defecations per week and faecal incontinence >2 times/week | Rome III Criteria | PEG 4000 PEG 4000 + probiotic mixture (Bifidobacteria breve + longum) | 28 27 | 7.1±2.5 7.4±2.8 | 53.6 51.9 | 8 | 12 | Italy |
| [62] | not mentioned | Rome III Criteria | lactulose (1 mL/kg/d) plus Protexin* lactulose (1 mL/kg/d) plus placebo | 24 24 | 6.1±2.4 6.3±1.9 | 41.7 58.3 | 4 | 4 | Iran |
| [63] | < 3 defecations per week and faecal incontinence >1 time/week | Rome III Criteria | Probiotic (Bifidobacteria lactis DN-173 010 + Lactococcus cremoris) Placebo (milk) | 79 80 | 7.0±3.4 6.5±3.1 | 46.8 48.8 | 3 | 5 | Netherlands and Poland |
| [64] | not mentioned | Rome III Criteria | liquid paraffin Synbiotic^&^ Synbiotic^&^ + liquid paraffin | 29 31 37 | 6.9±2.4 6.2±1.9 5.9±2.2 | 55.2 51.6 51.4 | 4 | 4 | Iran |
| [65] | < 3 bowel movement per week at least 8 weeks | not mentioned | Lactobacillus casei rhamnosus, Lcr35 MgO Placebo | 18 18 9 | 3.1±1.2 2.7±1.2 2.9±1.2 | 44.4 50.0 55.6 | 4 | 4 | Taiwan |
| [66] | < 3 bowel movement per week at least 12 weeks | not mentioned | Lactobacillus rhamnosus GG (ATCC 53103) + lactulose Placebo + lactulose | 43 41 | 6.6±3.9 5.4±3.0 | NA | 12 | 24 | Poland |

*: Protexin consists of Lactobacillus casei PXN 37, Lactobacillus rhamnosus PXN 54, Streptococcus thermophiles PXN 66, Bifidobacterium breve PXN 25, Lactobacillus acidophilus PXN 35, Bifidobacterium infantis (child specific) PXN 27, and Lactobacillus bulgaricus PXN 39, TVC

^&^: Synbiotic here consists of L. casei, L. rhamnosus, S. thermophilus, B. breve, L. acidophilus, B. infantis, fructooligosaccharide

^%^: study duration = treatment duration + post-treatment follow-up duration

Abbreviation: NA: not available; Tx: treatment

**eTable 5A:** SUCRA of the improvement in bowel movement or stool frequency

| Treatment | SUCRA |
| --- | --- |
| Protexin + lax | 93.3 |
| LacRh ATCC 53103 + lax | 78.9 |
| Synbiotic + lax | 68.5 |
| LacReuteri DSM 17938 + lax | 62.5 |
| LacCRh Lcr35 | 61.7 |
| LacRe DSM 17938 | 55.0 |
| Bif M-16/ BB536 + lax | 49.1 |
| lax | 46.2 |
| Synbiotic | 22.5 |
| Bif DN-173 010 + LacCr | 8.7 |
| Pla | 3.5 |

Sorted by order of mean rank of improvement in bowel movement or stool frequency (the former, the better improvement in bowel movement or stool frequency)

**eTable 5B:** SUCRA of the improvement in bowel movement or stool frequency: subgroup of single intervention

| Treatment | SUCRA |
| --- | --- |
| LacCRh Lcr35 | 83.8 |
| LacRe DSM 17938 | 75.0 |
| lax | 74.9 |
| Synbiotic | 36.3 |
| Bif DN-173 010 + LacCr | 19.8 |
| Pla | 10.2 |

Sorted by order of mean rank of improvement in bowel movement or stool frequency (the former, the better improvement in bowel movement or stool frequency)

**eTable 5C:** SUCRA of the rate of fecal incontinence

| Treatment | SUCRA |
| --- | --- |
| Protexin + lax | 86.7 |
| lax | 59.5 |
| LacReuteri DSM 17938 + lax | 26.9 |
| Bif M-16/ BB536 + lax | 26.9 |

Sorted by order of mean rank of rate of fecal incontinence (the former, the less rate of fecal incontinence)

**eTable 5D:** SUCRA of the drop-out rate

| Treatment | SUCRA |
| --- | --- |
| Synbiotic + lax | 81.1 |
| LacCRh Lcr35 | 58.4 |
| Synbiotic | 53.1 |
| Bif M-16/ BB536 + lax | 50.4 |
| Bif DN-173 010 + LacCr | 47.9 |
| Protexin + lax | 46.0 |
| Pla | 43.6 |
| lax | 43.6 |
| LacReuteri DSM 17938 + lax | 41.4 |
| LacRh ATCC 53103 + lax | 34.4 |

Sorted by order of mean rank of drop-out rate (the former, the less drop-out rate)

Abbreviation: 95% CI: 95% confidence interval; Bif DN-173 010 + LacCr: Probiotic (Bifidobacteria lactis DN-173 010 + Lactococcus cremoris); Bif M-16/ BB536 + lax: probiotic mixture (Bifidobacteria breve + longum) + laxative; ES: effect size; LacCRh Lcr35: Lactobacillus casei rhamnosus Lcr35; LacRe DSM 17938: Lactobacillus reuteri DSM 17938; LacReuteri DSM 17938 + lax: Lactobacillus reuteri DSM 17938 + laxative; LacRh ATCC 53103 + lax: Lactobacillus rhamnosus GG (ATCC 53103) + laxative; lax: laxative; NMA: network meta-analysis; OR: odds ratio; Pla: Placebo/control; Protexin + lax: Protexin (Lactobacillus casei PXN 37, Lactobacillus rhamnosus PXN 54, Streptococcus thermophiles PXN 66, Bifidobacterium breve PXN 25, Lactobacillus acidophilus PXN 35, Bifidobacterium infantis (child specific) PXN 27, and Lactobacillus bulgaricus PXN 39) + laxative; RCT: randomized controlled trial; SMD: standardized mean difference; SUCRA: surface under the cumulative ranking curve; Synbiotic + lax: Synbiotic (L. casei, L. rhamnosus, S. thermophilus, B. breve, L. acidophilus, B. infantis, fructooligosaccharide) + laxative

**eTable 6A:** League table of the improvement in bowel movement or stool frequency: subgroup of single intervention

| LacCRh Lcr35 |  | 0.14 (-0.52,0.79) |  |  | ***1.37 (0.52,2.23)** |
| --- | --- | --- | --- | --- | --- |
| 0.12 (-0.80,1.05) | LacRe DSM 17938 | 0.02 (-0.60,0.63) |  |  |  |
| 0.14 (-0.53,0.81) | 0.02 (-0.60,0.63) | lax | *0.52 (0.00,1.03) |  | ***1.24 (0.39,2.08)** |
| 0.65 (-0.21,1.52) | 0.53 (-0.27,1.33) | 0.52 (-0.00,1.04) | Synbiotic |  |  |
| ***1.27 (0.20,2.34)** | 1.15 (-0.19,2.49) | 1.13 (-0.01,2.28) | 0.62 (-0.68,1.92) | Bif DN-173 010 + LacCr | 0.10 (-0.21,0.41) |
| ***1.37 (0.32,2.43)** | 1.25 (-0.09,2.60) | ***1.24 (0.09,2.38)** | 0.72 (-0.58,2.02) | 0.10 (-0.22,0.42) | Pla |

Pairwise (upper-right portion) and network (lower-left portion) meta-analysis results are presented as estimate effect sizes for the outcome of improvement in bowel movement or stool frequency. Interventions are reported in order of mean ranking of bowel movement or stool frequency improvement, and outcomes are expressed as standardized mean difference (SMD) (95% confidence intervals). For the pairwise meta-analyses, SMD of more than 0 indicate that the treatment specified in the row got more improvement than that specified in the column. For the network meta-analysis (NMA), SMD of more than 0 indicate that the treatment specified in the column got more improvement than that specified in the row. Bold results marked with * indicate statistical significance.

**eTable 6B:** League table of the safety profile in aspect of rate of fecal incontinence

| Protexin + lax | ***0.10 (0.01,0.73)** |  |  |
| --- | --- | --- | --- |
| 0.10 (0.00,5.45) | lax | 0.54 (0.23,1.29) | 0.46 (0.04,5.43) |
| 0.06 (0.00,4.25) | 0.54 (0.22,1.36) | LacReuteri DSM 17938 + lax |  |
| 0.05 (0.00,6.46) | 0.46 (0.04,5.54) | 0.85 (0.06,11.56) | Bif M-16/ BB536 + lax |

Pairwise (upper-right portion) and network (lower-left portion) meta-analysis results are presented as estimate effect sizes for the outcome of safety profile in aspect of rate of fecal incontinence. Interventions are reported in order of mean ranking of safety, and outcomes are expressed as odds ratio (OR) (95% confidence intervals). For the pairwise meta-analyses, OR of less than 1 indicate that the treatment specified in the row got more safety than that specified in the column. For the network meta-analysis (NMA), OR of less than 1 indicate that the treatment specified in the column got more safety than that specified in the row. Bold results marked with * indicate statistical significance.

**eTable 6C:** League table of the tolerability in aspect of drop-out rate

| Synbiotic + lax |  | 0.16 (0.01,3.40) |  |  |  |  | 0.10 (0.01,2.04) |  |  |
| --- | --- | --- | --- | --- | --- | --- | --- | --- | --- |
| 0.21 (0.00,42.46) | LacCRh Lcr35 |  |  |  |  | 0.47 (0.03,8.52) | 0.47 (0.04,5.71) |  |  |
| 0.16 (0.00,8.38) | 0.73 (0.01,78.54) | Synbiotic |  |  |  |  | 0.64 (0.12,3.53) |  |  |
| 0.15 (0.00,23.02) | 0.71 (0.01,80.57) | 0.96 (0.01,76.31) | Bif M-16/ BB536 + lax |  |  |  | 0.67 (0.10,4.35) |  |  |
| 0.12 (0.00,49.21) | 0.56 (0.00,65.58) | 0.77 (0.00,183.54) | 0.80 (0.00,201.37) | Bif DN-173 010 + LacCr |  | 0.83 (0.24,2.85) |  |  |  |
| 0.10 (0.00,13.51) | 0.47 (0.00,46.89) | 0.64 (0.01,43.90) | 0.67 (0.01,48.94) | 0.83 (0.00,186.81) | Protexin + lax |  | 1.00 (0.22,4.47) |  |  |
| 0.10 (0.00,20.47) | 0.47 (0.01,21.88) | 0.64 (0.01,70.60) | 0.67 (0.01,78.15) | 0.83 (0.05,13.77) | 1.00 (0.01,102.43) | Pla | 1.00 (0.08,12.76) |  |  |
| 0.10 (0.00,5.10) | 0.47 (0.01,16.33) | 0.64 (0.03,13.46) | 0.67 (0.03,15.41) | 0.83 (0.01,78.84) | 1.00 (0.05,18.76) | 1.00 (0.03,35.97) | lax | 0.92 (0.10,8.06) | 0.60 (0.13,2.69) |
| 0.09 (0.00,8.20) | 0.43 (0.01,27.66) | 0.59 (0.01,24.78) | 0.61 (0.01,27.87) | 0.76 (0.00,118.37) | 0.92 (0.02,35.28) | 0.92 (0.01,60.60) | 0.92 (0.10,8.06) | LacReuteri DSM 17938 + lax |  |
| 0.06 (0.00,8.11) | 0.28 (0.00,28.17) | 0.38 (0.01,26.37) | 0.40 (0.01,29.40) | 0.50 (0.00,112.19) | 0.60 (0.01,37.95) | 0.60 (0.01,61.52) | 0.60 (0.03,11.27) | 0.65 (0.02,25.18) | LacRh ATCC 53103 + lax |

Pairwise (upper-right portion) and network (lower-left portion) meta-analysis results are presented as estimate effect sizes for the outcome of tolerability in aspect of drop-out rate. Interventions are reported in order of mean ranking of tolerability, and outcomes are expressed as odds ratio (OR) (95% confidence intervals). For the pairwise meta-analyses, OR of less than 1 indicate that the treatment specified in the row got more tolerability than that specified in the column. For the network meta-analysis (NMA), OR of less than 1 indicate that the treatment specified in the column got more tolerability than that specified in the row. Bold results marked with * indicate statistical significance.

Abbreviation: 95% CI: 95% confidence interval; Bif DN-173 010 + LacCr: Probiotic (Bifidobacteria lactis DN-173 010 + Lactococcus cremoris); Bif M-16/ BB536 + lax: probiotic mixture (Bifidobacteria breve + longum) + laxative; ES: effect size; LacCRh Lcr35: Lactobacillus casei rhamnosus Lcr35; LacRe DSM 17938: Lactobacillus reuteri DSM 17938; LacReuteri DSM 17938 + lax: Lactobacillus reuteri DSM 17938 + laxative; LacRh ATCC 53103 + lax: Lactobacillus rhamnosus GG (ATCC 53103) + laxative; lax: laxative; NMA: network meta-analysis; OR: odds ratio; Pla: Placebo/control; Protexin + lax: Protexin (Lactobacillus casei PXN 37, Lactobacillus rhamnosus PXN 54, Streptococcus thermophiles PXN 66, Bifidobacterium breve PXN 25, Lactobacillus acidophilus PXN 35, Bifidobacterium infantis (child specific) PXN 27, and Lactobacillus bulgaricus PXN 39) + laxative; RCT: randomized controlled trial; SMD: standardized mean difference; SUCRA: surface under the cumulative ranking curve; Synbiotic + lax: Synbiotic (L. casei, L. rhamnosus, S. thermophilus, B. breve, L. acidophilus, B. infantis, fructooligosaccharide) + laxative

**eTable 7: Inconsistency and heterogeneity of different intervention**

Part 1: design-by-treatment and loop inconsistency model

| Inconsistency model | chi^2^ | *p* value of Prob>chi^2^ |
| --- | --- | --- |
| Bowel movement or stool frequency | | |
| design-by-treatment | 0.16 | 0.6902 |
| loop inconsistency | 0.16 | 0.6902 |
| Fecal incontinence | | |
| design-by-treatment | 0.37 | 0.5430 |
| loop inconsistency | 0.37 | 0.5430 |
| Drop-out rate | | |
| design-by-treatment | 0.01 | 0.9381 |
| loop inconsistency | 0.01 | 0.9381 |

Part 2: side-splitting inconsistency model:

Part of changes in bowel movement or stool frequency

| Side | symmetric | | nosymmetric | | Treatments used | |
| --- | --- | --- | --- | --- | --- | --- |
|  | P>z | tau | P>z | tau |  |  |
| A D | . | . | . | . | A (reference): | Pla |
| A G * | 0.991 | 1.11E-07 | . | . | B: | Protexin + lax |
| A H * | 0.991 | 1.05E-07 | 0.991 | 1.36E-07 | C: | Bif M-16/ BB536 + lax |
| B H * | 0.996 | 1.44E-07 | 0.996 | 3.42E-08 | D: | Bif DN-173 010 + LacCr |
| C H * | 0.997 | 3.59E-08 | 0.997 | 7.49E-07 | E: | LacRh ATCC 53103 + lax |
| E H * | 0.997 | 1.00E-07 | 0.996 | 4.70E-08 | F: | LacRe DSM 17938 |
| F H * | 0.69 | 4.36E-08 | 0.69 | 1.15E-07 | G: | LacCRh Lcr35 |
| F I * | 0.69 | 4.04E-08 | 0.69 | 2.81E-08 | H: | lax |
| G H * | 0.991 | 4.14E-08 | 0.991 | 1.36E-07 | I: | LacReuteri DSM 17938 + lax |
| H I * | 0.995 | 9.76E-08 | . | . | J: | Synbiotic |
| H J * | 0.997 | 5.58E-08 | . | . | K: | Synbiotic + lax |
| H K * | 0.997 | 8.88E-08 | . | . |  |  |
| J K | . | . | . | . |  |  |

Part of fecal incontinence rate

| Side | symmetric | | nosymmetric | | Treatments used | |
| --- | --- | --- | --- | --- | --- | --- |
|  | P>z | tau | P>z | tau | A (reference): | Protexin + lax |
| A D * | 0.997 | 0.008206 | 0.997 | 0.008206 | B: | LacReuteri DSM 17938 + lax |
| B D * | 0.997 | 0.01222 | 0.997 | 0.000112 | C: | Bif M-16/ BB536 + lax |
| C D * | . | 0.003412 | . | 0.000656 | D: | lax |

Part of drop-out rate

| Side | symmetric | | nosymmetric | | Treatments used | |
| --- | --- | --- | --- | --- | --- | --- |
|  | P>z | tau | P>z | tau |  |  |
| A D | . | . | . | . | A (reference): | Pla |
| A G * | 1 | 1.286022 | . | . | B: | Protexin + lax |
| A H * | 1 | 1.286011 | 1 | 1.286016 | C: | Bif M-16/ BB536 + lax |
| B H * | 1 | 1.286006 | 1 | 1.286006 | D: | Bif DN-173 010 + LacCr |
| C H * | 1 | 1.286005 | 1 | 1.286005 | E: | LacRh ATCC 53103 + lax |
| E H * | 1 | 1.286023 | 1 | 1.286005 | F: | Synbiotic + lax |
| F H * | 1 | 1.286004 | 1 | 1.285997 | G: | LacCRh Lcr35 |
| F J * | 1 | 1 | . | . | H: | lax |
| G H * | 1 | 1.286017 | 1 | 1.286016 | I: | LacReuteri DSM 17938 + lax |
| H I * | 1 | 1.286009 | . | . | J: | Synbiotic |
| H J * | 1 | 1.28603 | . | . |  |  |

Part 3 Heterogeneity among the individual pair-wise comparison

| Comparison | | | Heterogeneity statistic | degrees of freedom | P | I squared** | Tau-squared | Treatments used | |
| --- | --- | --- | --- | --- | --- | --- | --- | --- | --- |
| H | - | C | 0 | 0 | . | .% | 0 | A (reference): | Pla |
| D | - | A | 0 | 0 | . | .% | 0 | B: | Protexin + lax |
| H | - | E | 0 | 0 | . | .% | 0 | C: | Bif M-16/ BB536 + lax |
| I | - | H | 0.23 | 2 | 0.892 | 0.00% | 0 | D: | Bif DN-173 010 + LacCr |
| G | - | A | 0 | 0 | . | .% | 0 | E: | LacRh ATCC 53103 + lax |
| H | - | A | 0 | 0 | . | .% | 0 | F: | LacRe DSM 17938 |
| H | - | G | 0 | 0 | . | .% | 0 | G: | LacCRh Lcr35 |
| H | - | F | 0 | 0 | . | .% | 0 | H: | lax |
| I | - | F | 0 | 0 | . | .% | 0 | I: | LacReuteri DSM 17938 + lax |
| J | - | H | 0 | 0 | . | .% | 0 | J: | Synbiotic |
| K | - | H | 0 | 0 | . | .% | 0 | K: | Synbiotic + lax |
| K | - | J | 0 | 0 | . | .% | 0 |  |  |
| H | - | B | 0 | 0 | . | .% | 0 |  |  |
| - |  |  | . | . | . | .% | . |  |  |

**eTable 8: Estimated between-studies standard deviation of different outcome**

| Outcome | Estimated between-studies standard deviation |
| --- | --- |
| Bowel movement or stool frequency | 4.370e-06 |
| Fecal incontinence | 0.00390622 |
| Drop-out rate | 1.2860007 |

**eTable 9: Quality of evidence for primary outcome: Change in tinnitus severity**

| Comparisons | Direct evidence | | Indirect evidence | | Network meta-analysis | |
| --- | --- | --- | --- | --- | --- | --- |
|  | Standardized mean difference (95% CI) | The final rating of direct evidence | Co-efficiency (Standard error) | The final rating of indirect evidence | Standardized mean difference (95% CI) | Overall quality of evidence |
| Protexin + lax vs LacRh ATCC 53103 + lax |  |  |  |  | 0.29 (-0.43,1.02) | ⨁⨁◯◯ low |
| Protexin + lax vs Synbiotic + lax |  |  |  |  | 0.43 (-0.33,1.19) | ⨁◯◯◯ very low |
| Protexin + lax vs LacReuteri DSM 17938 + lax |  |  |  |  | 0.48 (-0.16,1.13) | ⨁⨁◯◯ low |
| Protexin + lax vs LacCRh Lcr35 |  |  |  |  | 0.50 (-0.37,1.38) | ⨁◯◯◯ very low |
| Protexin + lax vs LacRe DSM 17938 |  |  |  |  | 0.57 (-0.23,1.37) | ⨁◯◯◯ very low |
| Protexin + lax vs Bif M-16/ BB536 + lax |  |  |  |  | 0.64 (-0.15,1.42) | ⨁◯◯◯ very low |
| Protexin + lax vs lax | ***0.64 (0.06,1.22)** | ⨁⨁⨁◯ medium | 2.47 (631.63) | ⨁◯◯◯ very low | ***0.64 (0.06,1.22)** | ⨁⨁⨁⨁ high |
| Protexin + lax vs Synbiotic |  |  |  |  | ***1.06 (0.29,1.84)** | ⨁⨁◯◯ low |
| Protexin + lax vs Bif DN-173 010 + LacCr |  |  |  |  | ***1.77 (0.70,2.84)** | ⨁⨁◯◯ low |
| Protexin + lax vs Pla |  |  |  |  | ***1.87 (0.85,2.90)** | ⨁⨁◯◯ low |
| LacRh ATCC 53103 + lax vs Synbiotic + lax |  |  |  |  | 0.14 (-0.51,0.79) | ⨁⨁◯◯ low |
| LacRh ATCC 53103 + lax vs LacReuteri DSM 17938 + lax |  |  |  |  | 0.19 (-0.32,0.70) | ⨁⨁◯◯ low |
| LacRh ATCC 53103 + lax vs LacCRh Lcr35 |  |  |  |  | 0.21 (-0.58,0.99) | ⨁◯◯◯ very low |
| LacRh ATCC 53103 + lax vs LacRe DSM 17938 |  |  |  |  | 0.28 (-0.43,0.98) | ⨁⨁◯◯ low |
| LacRh ATCC 53103 + lax vs Bif M-16/ BB536 + lax |  |  |  |  | 0.35 (-0.34,1.03) | ⨁⨁◯◯ low |
| LacRh ATCC 53103 + lax vs lax | 0.35 (-0.09,0.78) | ⨁⨁◯◯ low | 2.48 (646.95) | ⨁◯◯◯ very low | 0.35 (-0.09,0.78) | ⨁⨁⨁◯ medium |
| LacRh ATCC 53103 + lax vs Synbiotic |  |  |  |  | ***0.77 (0.10,1.44)** | ⨁⨁◯◯ low |
| LacRh ATCC 53103 + lax vs Bif DN-173 010 + LacCr |  |  |  |  | ***1.48 (0.48,2.48)** | ⨁⨁◯◯ low |
| LacRh ATCC 53103 + lax vs Pla |  |  |  |  | ***1.58 (0.63,2.53)** | ⨁⨁◯◯ low |
| Synbiotic + lax vs LacReuteri DSM 17938 + lax |  |  |  |  | 0.05 (-0.51,0.61) | ⨁⨁◯◯ low |
| Synbiotic + lax vs LacCRh Lcr35 |  |  |  |  | 0.07 (-0.75,0.88) | ⨁◯◯◯ very low |
| Synbiotic + lax vs LacRe DSM 17938 |  |  |  |  | 0.14 (-0.60,0.87) | ⨁⨁◯◯ low |
| Synbiotic + lax vs Bif M-16/ BB536 + lax |  |  |  |  | 0.21 (-0.51,0.92) | ⨁⨁◯◯ low |
| Synbiotic + lax vs lax | 0.21 (-0.28,0.69) | ⨁⨁◯◯ low | -2.16 (567.30) | ⨁◯◯◯ very low | 0.21 (-0.28,0.69) | ⨁⨁⨁◯ medium |
| Synbiotic + lax vs Synbiotic | ***0.63 (0.15,1.12)** | ⨁⨁⨁◯ medium |  |  | ***0.63 (0.15,1.12)** | ⨁⨁⨁◯ medium |
| Synbiotic + lax vs Bif DN-173 010 + LacCr |  |  |  |  | ***1.34 (0.32,2.36)** | ⨁⨁◯◯ low |
| Synbiotic + lax vs Pla |  |  |  |  | ***1.44 (0.47,2.42)** | ⨁⨁◯◯ low |
| LacReuteri DSM 17938 + lax vs LacCRh Lcr35 |  |  |  |  | 0.02 (-0.69,0.73) | ⨁⨁◯◯ low |
| LacReuteri DSM 17938 + lax vs LacRe DSM 17938 | 0.03 (-0.60,0.65) | ⨁◯◯◯ very low | 0.31 (0.63) | ⨁⨁◯◯ low | 0.08 (-0.47,0.64) | ⨁⨁⨁◯ medium |
| LacReuteri DSM 17938 + lax vs Bif M-16/ BB536 + lax |  |  |  |  | 0.15 (-0.44,0.75) | ⨁⨁◯◯ low |
| LacReuteri DSM 17938 + lax vs lax | 0.15 (-0.12,0.43) | ⨁⨁◯◯ low | -2.48 (466.36) | ⨁◯◯◯ very low | 0.15 (-0.12,0.43) | ⨁⨁⨁◯ medium |
| LacReuteri DSM 17938 + lax vs Synbiotic |  |  |  |  | ***0.58 (0.00,1.16)** | ⨁⨁◯◯ low |
| LacReuteri DSM 17938 + lax vs Bif DN-173 010 + LacCr |  |  |  |  | ***1.29 (0.35,2.23)** | ⨁⨁◯◯ low |
| LacReuteri DSM 17938 + lax vs Pla |  |  |  |  | ***1.39 (0.50,2.28)** | ⨁⨁◯◯ low |
| LacCRh Lcr35 vs LacRe DSM 17938 |  |  |  |  | 0.07 (-0.79,0.92) | ⨁◯◯◯ very low |
| LacCRh Lcr35 vs Bif M-16/ BB536 + lax |  |  |  |  | 0.14 (-0.70,0.98) | ⨁◯◯◯ very low |
| LacCRh Lcr35 vs lax | 0.14 (-0.52,0.79) | ⨁◯◯◯ very low | -2.80 (238.87) | ⨁◯◯◯ very low | 0.14 (-0.52,0.79) | ⨁⨁⨁◯ medium |
| LacCRh Lcr35 vs Synbiotic |  |  |  |  | 0.56 (-0.27,1.39) | ⨁◯◯◯ very low |
| LacCRh Lcr35 vs Bif DN-173 010 + LacCr |  |  |  |  | ***1.27 (0.36,2.18)** | ⨁⨁◯◯ low |
| LacCRh Lcr35 vs Pla | ***1.37 (0.52,2.23)** | ⨁⨁⨁◯ medium | -1.29 (242.95) | ⨁◯◯◯ very low | ***1.37 (0.52,2.23)** | ⨁⨁⨁⨁ high |
| LacRe DSM 17938 vs Bif M-16/ BB536 + lax |  |  |  |  | 0.07 (-0.69,0.84) | ⨁◯◯◯ very low |
| LacRe DSM 17938 vs lax | 0.02 (-0.60,0.63) | ⨁◯◯◯ very low | -0.30 (0.64) | ⨁⨁◯◯ low | 0.07 (-0.48,0.62) | ⨁⨁⨁◯ medium |
| LacRe DSM 17938 vs Synbiotic |  |  |  |  | 0.50 (-0.26,1.25) | ⨁⨁◯◯ low |
| LacRe DSM 17938 vs Bif DN-173 010 + LacCr |  |  |  |  | ***1.20 (0.15,2.26)** | ⨁⨁◯◯ low |
| LacRe DSM 17938 vs Pla |  |  |  |  | ***1.31 (0.30,2.32)** | ⨁⨁◯◯ low |
| Bif M-16/ BB536 + lax vs lax | 0.00 (-0.53,0.53) | ⨁◯◯◯ very low | 2.47 (626.58) | ⨁◯◯◯ very low | -0.00 (-0.53,0.53) | ⨁⨁⨁◯ medium |
| Bif M-16/ BB536 + lax vs Synbiotic |  |  |  |  | 0.43 (-0.31,1.16) | ⨁⨁◯◯ low |
| Bif M-16/ BB536 + lax vs Bif DN-173 010 + LacCr |  |  |  |  | ***1.13 (0.09,2.18)** | ⨁⨁◯◯ low |
| Bif M-16/ BB536 + lax vs Pla |  |  |  |  | ***1.24 (0.24,2.23)** | ⨁⨁◯◯ low |
| lax vs Synbiotic | 0.43 (-0.08,0.94) | ⨁⨁◯◯ low | -2.79 (545.91) | ⨁◯◯◯ very low | 0.43 (-0.08,0.94) | ⨁⨁⨁◯ medium |
| lax vs Bif DN-173 010 + LacCr |  |  |  |  | ***1.13 (0.23,2.03)** | ⨁⨁◯◯ low |
| lax vs Pla | ***1.24 (0.39,2.08)** | ⨁⨁⨁◯ medium | -0.10 (118.79) | ⨁◯◯◯ very low | ***1.24 (0.39,2.08)** | ⨁⨁⨁⨁ high |
| Synbiotic vs Bif DN-173 010 + LacCr |  |  |  |  | 0.71 (-0.33,1.74) | ⨁◯◯◯ very low |
| Synbiotic vs Pla |  |  |  |  | 0.81 (-0.18,1.80) | ⨁◯◯◯ very low |
| Bif DN-173 010 + LacCr vs Pla | 0.10 (-0.21,0.41) | ⨁⨁◯◯ low |  |  | 0.10 (-0.21,0.41) | ⨁⨁⨁◯ medium |

We followed Cochrane Handbook for GRADE ratings in BMJ [67] and one important network meta-analysis in Lancet [68] for quality assessment

**References:**

1. Page MJ, McKenzie JE, Bossuyt PM, Boutron I, Hoffmann TC, Mulrow CD, et al. (2021) The PRISMA 2020 statement: an updated guideline for reporting systematic reviews. Bmj 372:n71. 10.1136/bmj.n71

2. Basturk A, Artan R, Yilmaz A (2016) Efficacy of synbiotic, probiotic, and prebiotic treatments for irritable bowel syndrome in children: A randomized controlled trial. The Turkish journal of gastroenterology : the official journal of Turkish Society of Gastroenterology 27:439-443. 10.5152/tjg.2016.16301

3. Chen K, Xin J, Zhang G, Xie H, Luo L, Yuan S, et al. (2020) A combination of three probiotic strains for treatment of acute diarrhoea in hospitalised children: an open label, randomised controlled trial. Benef Microbes 11:339-346. 10.3920/BM2020.0046

4. Escribano J, Ferre N, Gispert-Llaurado M, Luque V, Rubio-Torrents C, Zaragoza-Jordana M, et al. (2018) Bifidobacterium longum subsp infantis CECT7210-supplemented formula reduces diarrhea in healthy infants: a randomized controlled trial. Pediatr Res 83:1120-1128. 10.1038/pr.2018.34

5. Reyna-Figueroa J, Barron-Calvillo E, Garcia-Parra C, Galindo-Delgado P, Contreras-Ochoa C, Lagunas-Martinez A, et al. (2019) Probiotic Supplementation Decreases Chemotherapy-induced Gastrointestinal Side Effects in Patients With Acute Leukemia. J Pediatr Hematol Oncol 41:468-472. 10.1097/MPH.0000000000001497

6. Zaja O, Fiolic M, Cuk MC, Tiljak MK (2021) "The role of L. reuteri DSM17938 in nutritional recovery and treatment of constipation in children and adolescents with anorexia nervosa - a randomized, double blind, placebo controlled study". Clin Nutr ESPEN 46:47-53. 10.1016/j.clnesp.2021.08.016

7. Kong XJ, Wan G, Tian R, Liu S, Liu K, Clairmont C, et al. (2021) The Effects of Probiotic Supplementation on Anthropometric Growth and Gut Microbiota Composition in Patients With Prader-Willi Syndrome: A Randomized Double-Blinded Placebo-Controlled Trial. Front Nutr 8:587974. 10.3389/fnut.2021.587974

8. Garcia Contreras AA, Vasquez Garibay EM, Sanchez Ramirez CA, Fafutis Morris M, Delgado Rizo V (2020) Lactobacillus reuteri DSM 17938 and Agave Inulin in Children with Cerebral Palsy and Chronic Constipation: A Double-Blind Randomized Placebo Controlled Clinical Trial. Nutrients 1210.3390/nu12102971

9. Sanctuary MR, Kain JN, Chen SY, Kalanetra K, Lemay DG, Rose DR, et al. (2019) Pilot study of probiotic/colostrum supplementation on gut function in children with autism and gastrointestinal symptoms. PloS one 14:e0210064. 10.1371/journal.pone.0210064

10. Guerra PV, Lima LN, Souza TC, Mazochi V, Penna FJ, Silva AM, et al. (2011) Pediatric functional constipation treatment with Bifidobacterium-containing yogurt: a crossover, double-blind, controlled trial. World journal of gastroenterology 17:3916-3921. 10.3748/wjg.v17.i34.3916

11. Coccorullo P, Strisciuglio C, Martinelli M, Miele E, Greco L, Staiano A (2010) Lactobacillus reuteri (DSM 17938) in infants with functional chronic constipation: a double-blind, randomized, placebo-controlled study. The Journal of pediatrics 157:598-602. 10.1016/j.jpeds.2010.04.066

12. Wegh CAM, Baaleman DF, Tabbers MM, Smidt H, Benninga MA (2022) Nonpharmacologic Treatment for Children with Functional Constipation: A Systematic Review and Meta-analysis. The Journal of pediatrics 240:136-149 e135. 10.1016/j.jpeds.2021.09.010

13. Zhou BG, Chen LX, Li B, Wan LY, Ai YW (2019) Saccharomyces boulardii as an adjuvant therapy for Helicobacter pylori eradication: A systematic review and meta-analysis with trial sequential analysis. Helicobacter 24:e12651. 10.1111/hel.12651

14. Guo Q, Goldenberg JZ, Humphrey C, El Dib R, Johnston BC (2019) Probiotics for the prevention of pediatric antibiotic-associated diarrhea. The Cochrane database of systematic reviews 4:CD004827. 10.1002/14651858.CD004827.pub5

15. Goldenberg JZ, Yap C, Lytvyn L, Lo CK, Beardsley J, Mertz D, et al. (2017) Probiotics for the prevention of Clostridium difficile-associated diarrhea in adults and children. The Cochrane database of systematic reviews 12:CD006095. 10.1002/14651858.CD006095.pub4

16. Huang R, Ning H, Shen M, Li J, Zhang J, Chen X (2017) Probiotics for the Treatment of Atopic Dermatitis in Children: A Systematic Review and Meta-Analysis of Randomized Controlled Trials. Front Cell Infect Microbiol 7:392. 10.3389/fcimb.2017.00392

17. Huang R, Hu J (2017) Positive Effect of Probiotics on Constipation in Children: A Systematic Review and Meta-Analysis of Six Randomized Controlled Trials. Front Cell Infect Microbiol 7:153. 10.3389/fcimb.2017.00153

18. Lu M, Yu S, Deng J, Yan Q, Yang C, Xia G, et al. (2016) Efficacy of Probiotic Supplementation Therapy for Helicobacter pylori Eradication: A Meta-Analysis of Randomized Controlled Trials. PloS one 11:e0163743. 10.1371/journal.pone.0163743

19. Hayes SR, Vargas AJ (2016) Probiotics for the Prevention of Pediatric Antibiotic-Associated Diarrhea. Explore (NY) 12:463-466. 10.1016/j.explore.2016.08.015

20. Schwenger EM, Tejani AM, Loewen PS (2015) Probiotics for preventing urinary tract infections in adults and children. The Cochrane database of systematic reviews:CD008772. 10.1002/14651858.CD008772.pub2

21. Goldenberg JZ, Lytvyn L, Steurich J, Parkin P, Mahant S, Johnston BC (2015) Probiotics for the prevention of pediatric antibiotic-associated diarrhea. The Cochrane database of systematic reviews:CD004827. 10.1002/14651858.CD004827.pub4

22. McFarland LV, Karakan T, Karatas A (2021) Strain-specific and outcome-specific efficacy of probiotics for the treatment of irritable bowel syndrome: A systematic review and meta-analysis. EClinicalMedicine 41:101154. 10.1016/j.eclinm.2021.101154

23. Harris RG, Neale EP, Ferreira I (2019) When poorly conducted systematic reviews and meta-analyses can mislead: a critical appraisal and update of systematic reviews and meta-analyses examining the effects of probiotics in the treatment of functional constipation in children. Am J Clin Nutr 110:177-195. 10.1093/ajcn/nqz071

24. Jin L, Deng L, Wu W, Wang Z, Shao W, Liu J (2018) Systematic review and meta-analysis of the effect of probiotic supplementation on functional constipation in children. Medicine 97:e12174. 10.1097/MD.0000000000012174

25. Mai TT, Thi Thu P, Thi Hang H, Trang TTT, Yui S, Shigehisa A, et al. (2021) Efficacy of probiotics on digestive disorders and acute respiratory infections: a controlled clinical trial in young Vietnamese children. Eur J Clin Nutr 75:513-520. 10.1038/s41430-020-00754-9

26. Hassanzad M, Maleki Mostashari K, Ghaffaripour H, Emami H, Rahimi Limouei S, Velayati AA (2019) Synbiotics and Treatment of Asthma: A Double-Blinded, Randomized, Placebo-Controlled Clinical Trial. Galen Med J 8:e1350. 10.31661/gmj.v8i0.1350

27. Basturk A, Isik I, Atalay A, Yilmaz A (2020) Investigation of the Efficacy of Lactobacillus rhamnosus GG in Infants With Cow's Milk Protein Allergy: a Randomised Double-Blind Placebo-Controlled Trial. Probiotics Antimicrob Proteins 12:138-143. 10.1007/s12602-019-9516-1

28. Kuizenga-Wessel S, Benninga MA, Tabbers MM (2015) Reporting outcome measures of functional constipation in children from 0 to 4 years of age. J Pediatr Gastroenterol Nutr 60:446-456. 10.1097/MPG.0000000000000631

29. Zhao HM, Ou-Yang HJ, Duan BP, Xu B, Chen ZY, Tang J, et al. (2014) [Clinical effect of triple therapy combined with Saccharomyces boulardii in the treatment of Helicobacter pylori infection in children]. Zhongguo Dang Dai Er Ke Za Zhi 16:230-233.

30. Tolone S, Pellino V, Vitaliti G, Lanzafame A, Tolone C (2012) Evaluation of Helicobacter Pylori eradication in pediatric patients by triple therapy plus lactoferrin and probiotics compared to triple therapy alone. Ital J Pediatr 38:63. 10.1186/1824-7288-38-63

31. Yang SS, Chiang IN, Lin CD, Chang SJ (2012) Advances in non-surgical treatments for urinary tract infections in children. World J Urol 30:69-75. 10.1007/s00345-011-0700-5

32. Savino F, Cordisco L, Tarasco V, Palumeri E, Calabrese R, Oggero R, et al. (2010) Lactobacillus reuteri DSM 17938 in infantile colic: a randomized, double-blind, placebo-controlled trial. Pediatrics 126:e526-533. 10.1542/peds.2010-0433

33. Puccio G, Cajozzo C, Meli F, Rochat F, Grathwohl D, Steenhout P (2007) Clinical evaluation of a new starter formula for infants containing live Bifidobacterium longum BL999 and prebiotics. Nutrition 23:1-8. 10.1016/j.nut.2006.09.007

34. Schrezenmeir J, Heller K, McCue M, Llamas C, Lam W, Burow H, et al. (2004) Benefits of oral supplementation with and without synbiotics in young children with acute bacterial infections. Clin Pediatr (Phila) 43:239-249. 10.1177/000992280404300305

35. Rahayu ES, Mariyatun M, Putri Manurung NE, Hasan PN, Therdtatha P, Mishima R, et al. (2021) Effect of probiotic Lactobacillus plantarum Dad-13 powder consumption on the gut microbiota and intestinal health of overweight adults. World journal of gastroenterology 27:107-128. 10.3748/wjg.v27.i1.107

36. D'Onofrio V, Del Chierico F, Belci P, Vernocchi P, Quagliariello A, Reddel S, et al. (2020) Effects of a Synbiotic Formula on Functional Bowel Disorders and Gut Microbiota Profile during Long-Term Home Enteral Nutrition (LTHEN): A Pilot Study. Nutrients 1310.3390/nu13010087

37. Madempudi RS, Neelamraju J, Ahire JJ, Gupta SK, Shukla VK (2020) Bacillus coagulans Unique IS2 in Constipation: A Double-Blind, Placebo-Controlled Study. Probiotics Antimicrob Proteins 12:335-342. 10.1007/s12602-019-09542-9

38. Ojetti V, Ianiro G, Tortora A, D'Angelo G, Di Rienzo TA, Bibbo S, et al. (2014) The effect of Lactobacillus reuteri supplementation in adults with chronic functional constipation: a randomized, double-blind, placebo-controlled trial. J Gastrointestin Liver Dis 23:387-391. 10.15403/jgld.2014.1121.234.elr

39. de Milliano I, Tabbers MM, van der Post JA, Benninga MA (2012) Is a multispecies probiotic mixture effective in constipation during pregnancy? 'A pilot study'. Nutr J 11:80. 10.1186/1475-2891-11-80

40. Vandenplas Y, Salvatore S (2016) Infant Formula with Partially Hydrolyzed Proteins in Functional Gastrointestinal Disorders. Nestle Nutr Inst Workshop Ser 86:29-37. 10.1159/000442723

41. Tabbers MM, de Milliano I, Roseboom MG, Benninga MA (2011) Is Bifidobacterium breve effective in the treatment of childhood constipation? Results from a pilot study. Nutr J 10:19. 10.1186/1475-2891-10-19

42. Bekkali NL, Bongers ME, Van den Berg MM, Liem O, Benninga MA (2007) The role of a probiotics mixture in the treatment of childhood constipation: a pilot study. Nutr J 6:17. 10.1186/1475-2891-6-17

43. Indrio F, Di Mauro A, Riezzo G, Civardi E, Intini C, Corvaglia L, et al. (2014) Prophylactic use of a probiotic in the prevention of colic, regurgitation, and functional constipation: a randomized clinical trial. JAMA pediatrics 168:228-233. 10.1001/jamapediatrics.2013.4367

44. Yang W, He T, Zhang W, Gu L, Tu R, Liu H (2019) Effectiveness and safety of lactobacilli in children with functional constipation: Study protocol for a meta-analysis and systematic review. Medicine 98:e15675. 10.1097/MD.0000000000015675

45. Daniel M, Szymanik-Grzelak H, Turczyn A, Panczyk-Tomaszewska M (2020) Lactobacillus rhamnosus PL1 and Lactobacillus plantarum PM1 versus placebo as a prophylaxis for recurrence urinary tract infections in children: a study protocol for a randomised controlled trial. BMC Urol 20:168. 10.1186/s12894-020-00723-1

46. Wojtyniak K, Szajewska H (2017) Systematic review: probiotics for functional constipation in children. Eur J Pediatr 176:1155-1162. 10.1007/s00431-017-2972-2

47. Scourboutakos MJ, Franco-Arellano B, Murphy SA, Norsen S, Comelli EM, L'Abbe MR (2017) Mismatch between Probiotic Benefits in Trials versus Food Products. Nutrients 910.3390/nu9040400

48. Koppen IJ, Benninga MA, Tabbers MM (2016) Is There A Role for Pre-, Pro- and Synbiotics in the Treatment of Functional Constipation in Children? A Systematic Review. J Pediatr Gastroenterol Nutr 63 Suppl 1:S27-35. 10.1097/MPG.0000000000001220

49. Barnes D, Yeh AM (2015) Bugs and Guts: Practical Applications of Probiotics for Gastrointestinal Disorders in Children. Nutr Clin Pract 30:747-759. 10.1177/0884533615610081

50. Cruchet S, Furnes R, Maruy A, Hebel E, Palacios J, Medina F, et al. (2015) The use of probiotics in pediatric gastroenterology: a review of the literature and recommendations by Latin-American experts. Paediatr Drugs 17:199-216. 10.1007/s40272-015-0124-6

51. Guandalini S, Cernat E, Moscoso D (2015) Prebiotics and probiotics in irritable bowel syndrome and inflammatory bowel disease in children. Benef Microbes 6:209-217. 10.3920/BM2014.0067

52. Urbanska M, Szajewska H (2014) The efficacy of Lactobacillus reuteri DSM 17938 in infants and children: a review of the current evidence. Eur J Pediatr 173:1327-1337. 10.1007/s00431-014-2328-0

53. Tabbers MM, Boluyt N, Berger MY, Benninga MA (2011) Nonpharmacologic treatments for childhood constipation: systematic review. Pediatrics 128:753-761. 10.1542/peds.2011-0179

54. Chmielewska A, Szajewska H (2010) Systematic review of randomised controlled trials: probiotics for functional constipation. World journal of gastroenterology 16:69-75. 10.3748/wjg.v16.i1.69

55. Szajewska H, Setty M, Mrukowicz J, Guandalini S (2006) Probiotics in gastrointestinal diseases in children: hard and not-so-hard evidence of efficacy. J Pediatr Gastroenterol Nutr 42:454-475. 10.1097/01.mpg.0000221913.88511.72

56. Vandenplas Y, Cruchet S, Faure C, Lee H, Di Lorenzo C, Staiano A, et al. (2014) When should we use partially hydrolysed formulae for frequent gastrointestinal symptoms and allergy prevention? Acta Paediatr 103:689-695. 10.1111/apa.12637

57. Wojtyniak K, Horvath A, Dziechciarz P, Szajewska H (2017) Lactobacillus casei rhamnosus Lcr35 in the Management of Functional Constipation in Children: A Randomized Trial. The Journal of pediatrics 184:101-105 e101. 10.1016/j.jpeds.2017.01.068

58. Kubota M, Ito K, Tomimoto K, Kanazaki M, Tsukiyama K, Kubota A, et al. (2020) Lactobacillus reuteri DSM 17938 and Magnesium Oxide in Children with Functional Chronic Constipation: A Double-Blind and Randomized Clinical Trial. Nutrients 1210.3390/nu12010225

59. Jadresin O, Sila S, Trivic I, Misak Z, Hojsak I, Kolacek S (2018) Lack of Benefit of Lactobacillus reuteri DSM 17938 as an Addition to the Treatment of Functional Constipation. J Pediatr Gastroenterol Nutr 67:763-766. 10.1097/MPG.0000000000002134

60. Wegner A, Banaszkiewicz A, Kierkus J, Landowski P, Korlatowicz-Bilar A, Wiecek S, et al. (2018) The effectiveness of Lactobacillus reuteri DSM 17938 as an adjunct to macrogol in the treatment of functional constipation in children. A randomized, double-blind, placebo-controlled, multicentre trial. Clin Res Hepatol Gastroenterol 42:494-500. 10.1016/j.clinre.2018.03.008

61. Russo M, Giugliano FP, Quitadamo P, Mancusi V, Miele E, Staiano A (2017) Efficacy of a mixture of probiotic agents as complementary therapy for chronic functional constipation in childhood. Ital J Pediatr 43:24. 10.1186/s13052-017-0334-3

62. Sadeghzadeh M, Rabieefar A, Khoshnevisasl P, Mousavinasab N, Eftekhari K (2014) The effect of probiotics on childhood constipation: a randomized controlled double blind clinical trial. Int J Pediatr 2014:937212. 10.1155/2014/937212

63. Tabbers MM, Chmielewska A, Roseboom MG, Crastes N, Perrin C, Reitsma JB, et al. (2011) Fermented milk containing Bifidobacterium lactis DN-173 010 in childhood constipation: a randomized, double-blind, controlled trial. Pediatrics 127:e1392-1399. 10.1542/peds.2010-2590

64. Khodadad A, Sabbaghian M (2010) Role of synbiotics in the treatment of childhood constipation: a double-blind randomized placebo controlled trial. Iran J Pediatr 20:387-392.

65. Bu LN, Chang MH, Ni YH, Chen HL, Cheng CC (2007) Lactobacillus casei rhamnosus Lcr35 in children with chronic constipation. Pediatr Int 49:485-490. 10.1111/j.1442-200X.2007.02397.x

66. Banaszkiewicz A, Szajewska H (2005) Ineffectiveness of Lactobacillus GG as an adjunct to lactulose for the treatment of constipation in children: a double-blind, placebo-controlled randomized trial. The Journal of pediatrics 146:364-369. 10.1016/j.jpeds.2004.10.022

67. Puhan MA, Schunemann HJ, Murad MH, Li T, Brignardello-Petersen R, Singh JA, et al. (2014) A GRADE working group approach for rating the quality of treatment effect estimates from network meta-analysis. Bmj 349:g5630. 10.1136/bmj.g5630

68. Cipriani A, Furukawa TA, Salanti G, Chaimani A, Atkinson LZ, Ogawa Y, et al. (2018) Comparative efficacy and acceptability of 21 antidepressant drugs for the acute treatment of adults with major depressive disorder: a systematic review and network meta-analysis. Lancet 391:1357-1366. 10.1016/S0140-6736(17)32802-7
